# Scalable set of reversible parity gates for integer factorization

**DOI:** 10.1038/s42005-023-01191-3

**Published:** 2023-04-17

**Authors:** Martin Lanthaler, Benjamin E. Niehoff, Wolfgang Lechner

**Affiliations:** 1 Institute for Theoretical Physics, University of Innsbruck, Innsbruck, Austria; 2 Parity Quantum Computing GmbH, Innsbruck, Austria

**Keywords:** Quantum information, Theoretical physics

## Abstract

Classical microprocessors operate on irreversible gates, that, when combined with AND, half-adder and full-adder operations, execute complex tasks such as multiplication of integers. We introduce parity versions of all components of a multiplication circuit. The parity gates are reversible quantum gates based on the recently introduced parity transformation and build on ground-space encoding of the corresponding gate logic. Using a quantum optimization heuristic, e.g., an adiabatic quantum computing protocol, allows one to quantum mechanically reverse the process of multiplication and thus factor integers, which has applications in cryptography. Our parity approach builds on nearest-neighbor constraints equipped with local fields, able to encode the logic of a binary multiplication circuit in a modular and scalable way.

## Abstract (web summary)

The authors present a method of prime factorization using quantum logic, based on parity-based gates. Using this approach, they formulate factorization as an optimization problem and show a quadratic advantage in the number of qubits required over other standard representations.

## Introduction

The inherent asymmetry in the complexity of multiplication and factorization has played a crucial role in cryptography and serves as the foundation for protocols such as RSA^1^. From the point of view of complexity theory, it is unlikely that the factoring problem is either in NP-complete or P, but it was proven that the problem lies in NP ∩ coNP^2^. As a search problem, factoring belongs to the class of total search problems TFNP and, more precisely, to PPA ∩ PPP under randomized reductions^3^.

On the other hand, Shor’s algorithm shows that factoring is an easy problem on quantum devices with an exponential speedup compared to any known classical algorithm^4,5^, i.e., factoring belongs to the complexity class BQP. However, due to the extensive requirements on the number of qubits and quality of gates, Shor’s algorithm is still limited to proof-of-concept demonstrations, far away from factoring numbers of magnitude used in real-world cryptosystems^6–11^.

In the framework of the adiabatic quantum computer, integer factorization is recast as unconstrained optimization that is solved using adiabatic quantum dynamics^12–14^. Experimentally, this approach was first validated for the small number 143 = 11 ⋅ 13 using NMR technology^15–17^. Subsequently, larger bi-primes were factorized^18–21^ using a novel approach based on multiplication tables^13^. In this approach, ancilla variables have to be introduced to mediate carry-overflows between different powers of two. Using ideas from algebraic geometry, i.e., Gröbner bases, the number of these auxiliary variables can be reduced by classical preprocessing, thereby enabling a demonstration of factorization up to 223.357^22^. Finally, simulations on D-Wave’s hybrid quantum/classical simulator qbsolv show, it is possible to factorize 1.005.973 on current hardware^23^, (While this manuscript was under revision, this record has been broken in ref. ^24^ and ref. ^25^ building on a variational approach.). All these methods, which are based on variants of the multiplication table, reduce the integer factorization problem to a quadratic unconstrained binary optimization (QUBO) problem involving ${{{{{{{\mathcal{O}}}}}}}}({\log }^{2}(n))$ logical qubits. To solve the optimization problem using adiabatic quantum computing techniques, the structure of the corresponding 2-local Hamiltonian must be mapped onto a connectivity graph available on hardware, e.g., via minor embedding^26,27^.

**Fig. 1:**
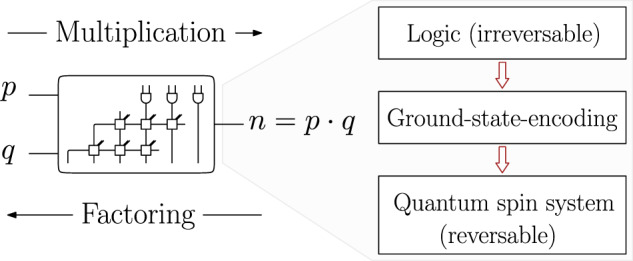
General idea. By introducing reversible gates, factorization is considered a reverse multiplication. The problem is reformulated as an optimization problem by encoding the logic in the ground space of a spin Hamiltonian.

In this article, we construct a novel reduction of the factorization problem to a parity-based spin model using a total of ${{{{{{{\mathcal{O}}}}}}}}({\log }^{2}(n))$ physical qubits and interaction strengths of order ${{{{{{{\mathcal{O}}}}}}}}(1)$. That’s a considerable improvement compared to previous approaches where ${{{{{{{\mathcal{O}}}}}}}}({\log }^{4}(n))$ physical qubits and interaction strengths ${{{{{{{\mathcal{O}}}}}}}}({\log }^{2}(n))$ are needed^13,19,28^. We propose to design a quantum factoring device by constructing a reversible version of a classical multiplication circuit built from basic AND, half-, and full-adder gates. This is accomplished by encoding each previously irreversible logic gate in the ground space of a spin Hamiltonian. By summing up all local “gate” Hamiltonians, we construct a spin glass model, which alongside constraints on input integers for multiplication or output integers for factorization, can perform either task [see Fig. 1]. The proposed Hamiltonians are tied to unit cells that can be arranged in a modular manner and are, therefore, easily scalable.

## Results

Instead of following the path from multiplication table to QUBO to hardware graph, we propose to design a special-purpose quantum device with a hardware graph that directly implements the logic of a binary multiplication circuit. The framework in which this is done, is based on a promising approach to encode optimization problems into a spin system using local fields and quasi-local 3- and 4-body parity constraints^29^. In this more general context, the work presented here explores what’s feasible within the parity framework, i.e., how the factoring problem can be tackled by building on parity ’plaquettes’ equipped with local fields.

### Overview

**Fig. 2:**
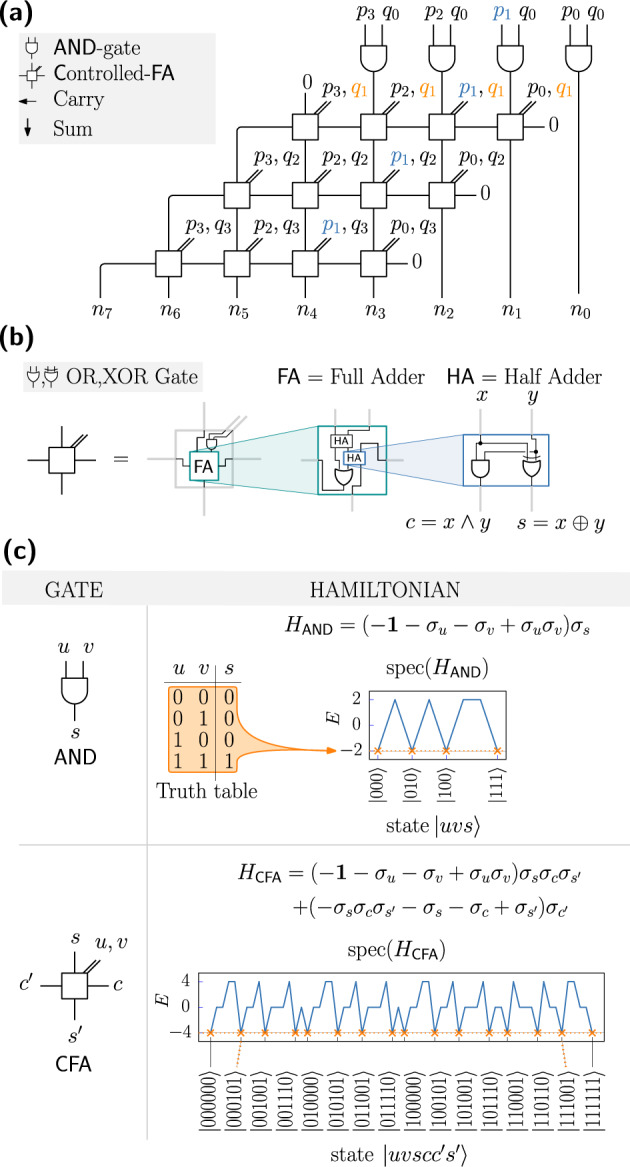
Boolean multiplier circuit and ground-state-encoding. **a** Since *p*_*i*_*q*_*j*_ = *p*_*i*_ ∧ *q*_*j*_, the partial products *p*_*i*_*q*_*j*_ can be formed using an AND gate. Subsequently, they can be summed up by full adders arranged on a 2D grid. Note that the gates share common inputs, as the values *p*_*i*_ and *q*_*j*_ repeat vertically or horizontally, respectively. **b** Full adders can be built from AND, OR, and XOR gates. **c** The AND gate can be encoded into the degenerated ground space of a Hamiltonian with four terms. Likewise, the controlled full adder can be encoded by a Hamiltonian consisting of eight terms.

It is straightforward to build a Boolean circuit that takes as input the binary representations of two numbers *p*, *q* and outputs their product *n*. As depicted in Fig. 2, such a circuit can be built from basic AND gates and full-adder (FA) gates. Subsection “Ground state spin logic” shows how the logic of the circuit is implemented within a spin system by designing a Hamiltonian whose ground states encode valid input-output relations of these gates. With this, the Hamiltonian ${H}_{{{{{{{{\rm{circuit}}}}}}}}}={\sum }_{\omega }{H}_{{\mathsf{AND}}}^{(\omega )}+{\sum }_{\omega }{H}_{{\mathsf{FA}}}^{(\omega )}$ has a ground space spanned by states obeying the correct multiplicative relationship. To single out one specific triple (*p*, *q*, *n*), one adds a term *H*_in_(*p*, *q*), assigning an energy penalty to all states not having *p* and *q* as their inputs. Hence, finding the ground state of *H*_product_ = *H*_circuit_ + *H*_in_(*p*, *q*) would solve the (easy) task of multiplying numbers *p* and *q*. However, the same approach is applicable in the case of factorization: The output number *n* can be fixed by adding a term *H*_factors_ = *H*_circuit_ + *H*_out_(*n*). With this, the challenging task of factorization can be implemented via optimization, and in particular via the approach of quantum optimization.

**Fig. 3:**
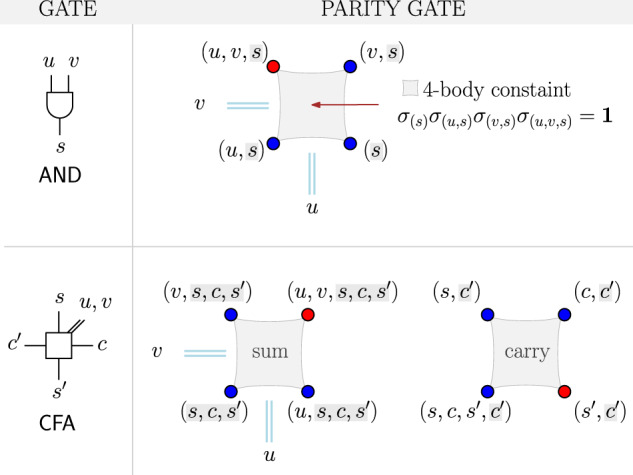
Parity gates. AND and CFA gates are implemented via ground state coding on four or eight qubits, respectively [see Fig. 2c]. While individual physical qubits encode parity information (denoted by the tuple, e.g., *σ*_(*u*, *v*, *s*)_), the gates are defined by local fields (red + 1 and blue − 1) and 4-body constraints ∏ω∈*σ*_*ω*_ = **1** as additional penalty term (highlighted by gray squares). Thus, the operator *H*_AND_ is implemented on a single parity plaquette. Conversely, the Hamiltonian *H*_CFA_ is implementable by means of two parity plaquettes denoted by “sum” and “carry''.

**Fig. 4:**
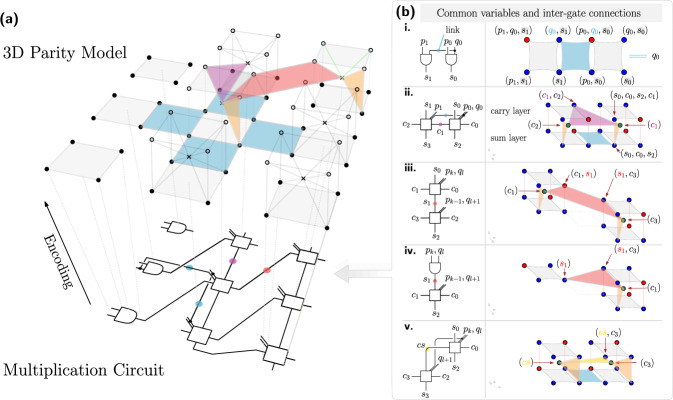
Blueprint for parity factorization - Three-dimensional layout. **a** The multiplication circuit is mapped onto an array of physical qubits arranged on three layers of a 3D lattice. Dots and crosses represent qubits on different layers while faces represent constraints [see Supplementary Figure 2 for the labeling of the qubits and Supplementary Fig. 3 for the plot including all constraints]. Beside boundaries, the whole architecture can be constructed from repeating unit cell consisting of nine qubits connected to neighboring cells via additional constraints (blue, red, violet, yellow, orange). For illustrative purposes, only a selected number of qubits and constraints are displayed. **b** Building instructions. Each connection between logical gates translates into 3-body or 4-body parity constraints (blue, red, violet, yellow). The device is programmable in the sense that a given bi-prime *n* can be encoded by imposing additional 2-body constraints (**a** green). Thus the ground state $\left\vert n=pq\right\rangle$ reveals the factors *p* and *q*.

**Fig. 5:**
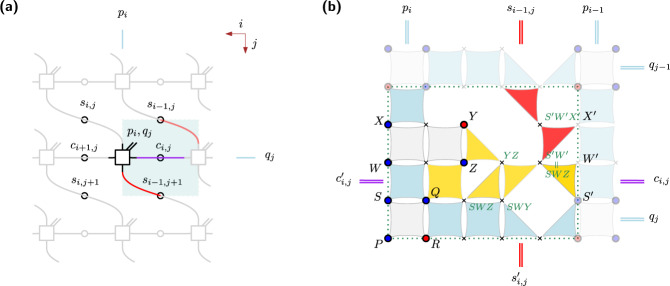
Blueprint for parity factorization - Planar layout. **a** Multiplication circuit as an array of CFA cells. Cells are enumerated by their inputs, *p*_*i*_ and *q*_*j*_. **b** Parity-based planar CFA unit cell. Compared to the 3D implementation, the unused space between primary plaquettes (*PQRS*)_*ij*_ is enlarged to embed the second primary plaquette (*WXYZ*)_*ij*_. Additional plaquettes transmit and process incoming and outgoing carry/sum variables. The multiplication circuit is then constructed by tiling the plane with unit cells, treating border cases differently, as shown in Supplementary Fig. 4.

In general, there exists a whole family of Hamiltonians able to encode the logic of the AND and the FA gates in its ground space. The choice made in subsection “Ground space spin logic” is motivated by the aspects of resources, i.e., the number of qubits and the number of interactions needed, and taking into account scalability. Instead of using these Hamiltonians directly, we use a parity representation of them as described in the subsection “Parity mapping”, which reduces the degree and amount of interactions needed [see Fig. 3]. In the “Assembling instructions” subsection, we present two possible ways to implement the inter-gate connections. While the first layout [see Fig. 4] is efficient in terms of the number of auxiliary qubits, its geometry could make it difficult to implement due to its 3D nature. Therefore, we propose a planar version that uses parity constraints on a two-dimensional grid at the expense of additional qubits [see Fig. 5]. Either way, the resulting Hamiltonian

1 $${H}_{{{{{{{{\rm{factors}}}}}}}}}^{{{{{{{{\rm{parity}}}}}}}}}:= {H}_{{{{{{{{\rm{circuit}}}}}}}}}^{{{{{{{{\rm{parity}}}}}}}}}+{H}_{{{{{{{{\rm{out}}}}}}}}}^{{{{{{{{\rm{parity}}}}}}}}}(n)$$

is geometrical local and consists of unit cells s.t. factoring bigger numbers can be done in a scalable and modular way by adding more of these unit cells. Finally, we show how to encode the desired bi-prime *n* by defining *H*_out_(*n*) in the parity picture ${H}_{{{{{{{{\rm{out}}}}}}}}}^{{{{{{{{\rm{parity}}}}}}}}}(n)$. The resulting construction provides a scalable, geometric local, and programmable Hamiltonian whose ground state encodes the factors *p* and *q* s.t. *n* = *p* ⋅ *q* holds. The ground state can be prepared using quantum heuristics, e.g., quantum annealing. In Supplementary Note 6, we offer a proof of principle by simulating the induced dynamics for a small instance.

### Notation

We make receptive use of diagonal Hamiltonians of form

2 $$H=\mathop{\sum}\limits_{i}{a}_{i}{\sigma }_{z}^{(i)}+\mathop{\sum}\limits_{ij}{a}_{ij}{\sigma }_{z}^{(i)}{\sigma }_{z}^{(j)}+\mathop{\sum}\limits_{ijk}{a}_{ijk}{\sigma }_{z}^{(i)}{\sigma }_{z}^{(j)}{\sigma }_{z}^{(k)}+\ldots .$$

with the Pauli *σ*_*z*_ defined by ${\sigma }_{z}:= \left\vert 0\right\rangle \,\left\langle 0\right\vert -\left\vert 1\right\rangle \,\left\langle 1\right\vert$. Since Hamiltonians of the form of Eq. (2) are classical, we like to slim down the notation and write *σ* ≡ *σ*_*z*_ s.t. *σ*_*i*_ denoted the Pauli-Z operator acting on qubits *i*. Terms *σ*_*i*_*σ*_*j*_ are used as a shorthand notation for the tensor product *σ*_*i*_ ⊗ *σ*_*j*_, where the subscript indicates which spin the operator acts. Natural numbers *n*, *p*, *q* are represented in binary as *n* ≡ *n*_*l*_. . . *n*_1_*n*_0_ via *n* = ∑_*i*_*n*_*i*_2^*i*^ and *n*_*i*_ ∈ {0, 1}. Throughout this document, we deal with irreversible classical gates and reversible (classical) gates encoded as the ground-state subspace of local spin Hamiltonians. The corresponding ground subspace is spanned by states defined by the logic of the classical gate and will be subsequently called the ’logical subspace’. At some point, it may be helpful to distinguish between the classical gate and a corresponding ground state implementation. Unless otherwise specified, the terminus gate should always refer to the classical gate, while corresponding parity implementations are denoted as ’parity gates’.

### Ground state spin logic

The idea behind ground-state spin logic, as described in^30^, is to embed a set of bit-strings ${{{{{{{\mathcal{S}}}}}}}}\subseteq {\{0,1\}}^{m}$ into the ground-state subspace of a spin Hamiltonian ${H}_{{{{{{{{\mathcal{S}}}}}}}}}$. Consider the AND gate, which defines four valid bit configurations (*u*, *v*, *s* = *u* ∧ *v*). The corresponding Hamiltonian *H*_AND_ is characterized by its degenerated lowest energy subspace

3 $${{{{{{{\mathcal{L}}}}}}}}({H}_{{\mathsf{AND}}})={{{{{{{\rm{span}}}}}}}}\{\left\vert 000\right\rangle ,\left\vert 010\right\rangle ,\left\vert 100\right\rangle ,\left\vert 111\right\rangle \}.$$

It is easy to find a whole family of Hamiltonians with the desired subspace defined by Eq. (3). One particular choice is given by

4 $${H}_{{\mathsf{AND}}}:= (-{{{{{{{\bf{1}}}}}}}}-{\sigma }_{u}-{\sigma }_{v}+{\sigma }_{u}{\sigma }_{v}){\sigma }_{s}.$$

We use this embedding because it has some desirable properties: The Hamiltonian consists of only four terms, the minimum number of terms required. The coupling strengths are − 1 or 1, and the spectrum of *H*_AND_ takes only two values { − 2, 2}, as shown in Fig. 2c. Furthermore, each of the indices occurs with even frequency (after expanding the expression the ’*u*’ and ’*v*’ occur twice and the ’*s*’ four times).

Similarly, the multiplicative relation between two integers can be encoded into the degenerated, low-energy ground space of a spin Hamiltonian. Figure 2a shows a possible circuit based on AND, half-adder, and full-adder gates. The AND implements the binary multiplication between bits *p*_*i*_ and *q*_*j*_ via the relation *p*_*i*_ ∧ *q*_*i*_ = *p*_*i*_*q*_*j*_, and further, the FA (full-adder) proceeds a previous carry overflow *c* and a sum *s* into a new sum and carry variable ${s}^{{\prime} }$ respectively ${c}^{{\prime} }$ (binary variables) such that

5 $$s+c+{p}_{i}\cdot {q}_{j}=2{c}^{{\prime} }+{s}^{{\prime} }$$

holds. See Fig. 2c and consult Supplementary Note 1 for further details.

Let us call the unit cell, build from an AND gate and a full-adder gate, a CFA-gate (**C**ontrolled-**F**ull**A**dder) [see Fig. 2b]. W.l.o.g. the whole circuit can be constructed from CFA gates arranged on a 2D grid with horizontal, vertical, and diagonal connections. The CFA gate operates on six bits, of which four act as input bits. The input *q*_*j*_ can be interpreted as control: When set to zero the gate acts as a half adder - processing the carry and sum inputs only. When set to one, it includes the *p*_*i*_ bit - acting as a full-adder. There are 16 valid bit configurations. These bit-strings can be encoded into the ground space of a spin Hamiltonian consisting of eight terms:

6 $${H}_{{\mathsf{CFA}}}:= 	 \,(-{{{{{{{\bf{1}}}}}}}}-{\sigma }_{u}-{\sigma }_{v}+{\sigma }_{u}{\sigma }_{v}){\sigma }_{s}{\sigma }_{c}{\sigma }_{{s}^{{\prime} }}\\ 	 + (-{\sigma }_{s}{\sigma }_{c}{\sigma }_{{s}^{{\prime} }}-{\sigma }_{s}-{\sigma }_{c}+{\sigma }_{{s}^{{\prime} }}){\sigma }_{{c}^{{\prime} }}.$$

Here, *s* and ${s}^{{\prime} }$ are the sum in- and outputs while *c* and ${c}^{{\prime} }$ denote the carry in-, and outputs, respectively. The two remaining nodes, *u* and *v,* are the inputs of the AND gate. Figure 2(c) shows the spectrum of this Hamiltonian. The ground-state manifold has energy − 4 while the other states have an energy of 0 or + 4, respectively. Remarkably, the first four terms are very similar to an AND gate. A formal replacement of the term *σ*_*s*_ with the product ${\sigma }_{s}{\sigma }_{c}{\sigma }_{{s}^{{\prime} }}$ yields them from the AND gate Hamiltonian Eq. (4). Similar to the AND gate, this part of the Hamiltonian matches the parity of $(s,c,{s}^{{\prime} })$ according to the input on qubits *u* and *v* following the logic of an AND gate. Since they do not interact with the carry output ${c}^{{\prime} }$, we call these terms the ’sum terms’. However, without the ’carry terms’ - the other four terms - the ground subspace would be 32-fold degenerate, which allows all possible states without fixing ${c}^{{\prime} }$. Adding these carry terms removes this degeneracy and penalizes all states not having the correct carry bit.

Again, a whole family of Hamiltonians can encode the CFA logic. However, this Hamiltonian is desirable since (after expansion) it contains every index $u,v,s,c,{c}^{{\prime} }$ and ${s}^{{\prime} }$ an even number of times. We will now elaborate on why this property is crucial for our construction.

### Parity mapping

Recently, a translation from an all-to-all 2-local spin model to a quasi-local model on a 2D lattice was introduced^31^. This approach can be generalized to include higher-order terms^29^. For each term appearing in Eq. (4) and Eq. (6) we introduce a physical spin. Imposing

7 $$\left\langle \right.{\sigma }_{(i,j,k,...)}{\rangle }_{{{{{{{{\rm{phys}}}}}}}}}=\left\langle \right.{\sigma }_{i}{\sigma }_{j}{\sigma }_{k}...{\rangle }_{{{{{{{{\rm{logi}}}}}}}}},$$

the physical spin, subscribed by (*i*, *j*, *k*, . . . ), is set to ${\left\vert 0\right\rangle }_{{{{{{{{\rm{phys}}}}}}}}}$ if the logical system is in a state with an even number of $\left\vert 1\right\rangle$’s on positions *i*, *j*, *k*, . . .   and set to ${\left\vert 1\right\rangle }_{{{{{{{{\rm{phys}}}}}}}}}$ otherwise. The newly introduced variable thus encodes the parity of a given subset of logical spins in a given state. In that sense, a 2-local term *σ*_*i*_*σ*_*j*_ - as discussed in^31^ - only discriminate states according to their relative orientation between the spins s.t. parallel aligned spins map onto ${\left\vert 0\right\rangle }_{{{{{{{{\rm{phys}}}}}}}}}$, and the subspace spanned by anti-parallel spins is mapped onto ${\left\vert 1\right\rangle }_{{{{{{{{\rm{phys}}}}}}}}}$.

In the case of the AND gate, the Hamiltonian Eq. (4) has four terms. Consequently, we introduce four physical qubits. Let’s call them *O* ≔ *σ*_*s*_, *U* ≔ *σ*_(*u*, *s*)_, *V* ≔ *σ*_(*v*, *s*)_ and *B* ≔ *σ*_(*u*, *v*, *s*)_. Rewritten in these new variables, the Hamiltonian *H*_AND_ reduces to a sum of local fields. Of special interest is the subspace of all physical states having a logical counterpart, i.e., they can be obtained by translating a logical state into the new variables. All such states from the logical subspace, obey the parity condition

8 $$OUVB={({\sigma }_{u})}^{2}{({\sigma }_{v})}^{2}{({\sigma }_{s})}^{4}={{{{{{{\bf{1}}}}}}}}.$$

This holds because of our choice for the AND gate encoding Eq. (4) and the identity ${({\sigma }_{i})}^{2}={{{{{{{\bf{1}}}}}}}}$. Consequently, only every second basis state belongs to the logical subspace. This is reasonable since there are eight possible bit configurations (*u*, *v*, *s*), i.e., the Hilbertspace of three qubits is 2^3^ = 8 dimensional, and we map these to a system with four physical qubits with a total of 16 dimensions. The addition of a penalty term splits the set of physical states according to their parity and favors the logical subspace energetically. Summarizing, the parity version of the AND gates writes as

9 $${H}_{{\mathsf{AND}}}^{{{{{{{{\rm{parity}}}}}}}}}: = \;	{H}_{{\mathsf{AND}}}^{{{{{{{{\rm{local\ fields}}}}}}}}}+{H}_{{\mathsf{AND}}}^{{{{{{{{\rm{penalty}}}}}}}}}\\ = \,	-O-U-V+B-kOUVB,$$

where *k* > 1 [see Supplementary Note 4 for a detailed discussion of constraint strengths]. The corresponding qubits are arranged on a plaquette of four s.t. the 4-local penalty term is also local in a geometrical sense. While the output is directly accessible *O* = *σ*_*s*_, the inputs *u* and *v* are encoded between horizontally or vertically adjacent qubits. Restricted to the subspace of states satisfying the parity constraint, we have the identities *BU* = *VO* = *σ*_*v*_ and *BV* = *UO* = *σ*_*u*_. If arranged according to Fig. 3, the plaquette encodes the variable *v* between horizontally adjacent qubits, while *u* is encoded vertically. In this sense, the plaquette acts as a wire, where information related to the variables *u* and *v* flow in perpendicular directions. These wires can be lengthened in one direction by extending them with additional plaquettes, as shown in Fig. 4(b.i). Here, the vertical encoded variable is *q*_0_ and is transmitted via

10 $${\sigma }_{{q}_{0}} = {\sigma }_{{s}_{0}}{\sigma }_{({q}_{0},{s}_{0})}={\sigma }_{({s}_{0},{p}_{0})}{\sigma }_{({p}_{0},{q}_{0},{s}_{0})} \\ ={\sigma }_{({s}_{1})}{\sigma }_{({p}_{0},{q}_{0},{s}_{1})}={\sigma }_{({p}_{1},{s}_{1})}{\sigma }_{({p}_{1},{p}_{0},{q}_{0},{s}_{1})},$$

which is true for all states in the logical subspace.

In the following section, we focus on the controlled full adder. As pointed out, the four sum-terms in *H*_CFA_ do not differ conceptually from the AND gate encoding Eq. (4). Consequently, we can assign them to a 4-body plaquette with the corresponding parity penalty. We call this the ’sum plaquette’. The corresponding variables should be denoted as *S* = *σ*_(⊢)_, *P* = *σ*_(*u*, ⊢)_, *Q* = *σ*_(*v*, ⊢)_ and *R* = *σ*_(*u*, *v*, ⊢)_ up to formal substitution $\vdash \,:= s,c,{s}^{{\prime} }$. Due to construction, the other four terms can be identified with a physical spin each s.t. ${\sigma }_{(s,c,{s}^{{\prime} },{c}^{{\prime} })}{\sigma }_{(s,{c}^{{\prime} })}{\sigma }_{(c,{c}^{{\prime} })}{\sigma }_{({s}^{{\prime} },{c}^{{\prime} })}={{{{{{{\bf{1}}}}}}}}$ if, and only if they are connected via the parity mapping Eq. (7) to a logical state. Let us denote these spin variables with *W*, *X*, *Y,* and *Z* in the order ${\sigma }_{(s,c,{s}^{{\prime} },{c}^{{\prime} })},{\sigma }_{(s,{c}^{{\prime} })},{\sigma }_{(c,{c}^{{\prime} })}$ and ${\sigma }_{({s}^{{\prime} },{c}^{{\prime} })}$. These terms can be combined in a second plaquette, which we call the ‘carry plaquette’ [see Fig. 3]. Consequently, the parity version of the CFA Hamiltonian introduced in Eq. (6) is given by

11 $${H}_{{\mathsf{CFA}}}^{{{{{{{{\rm{parity}}}}}}}}}:= \;	{H}_{{\mathsf{CFA}}}^{{{{{{{{\rm{local\ fields}}}}}}}}}+{H}_{{\mathsf{CFA}}}^{{{{{{{{\rm{penalty}}}}}}}}}\\ = \,	-S-P-Q+R-W-X-Y+Z\\ 	 - kSPQR-kWXYZ,$$

with penalty *k* > 1. To contrast Eq. (11) to a direct implementation of *H*_CFA_, we only need 1-local fields and two 4-body terms instead of three 2-body, one 3-body, three times a 4-body and one 5-body term in Eq. (6). Restricted to the logical subspace we have *SPQR* = **1**, *WXYZ* = **1** and the original variables can be calculated from the new ones, as given by the expressions in Table 1.

**Table 1:** Variable transformation.

*σ*_*u*_ = *RQ* = *PS*,	*σ*_*v*_ = *RP* = *QS*
*σ*_*c*_ = *SWZ*,	*σ*_*s*_ = *SWX*
${\sigma }_{{c}^{{\prime} }}=SW$,	${\sigma }_{{s}^{{\prime} }}=SWY$.

Input and output variables of CFA gates are encoded as products of physical spin operators.

### Assembling instructions

In this section, we present two different ways to construct the multiplication circuit from primary parity gates. The computational subspace is a degenerated stabilizer space spanned by states encoding valid multiplications of *L*_*p*_-bit times *L*_*q*_-bit integers. For simplicity, we assume *L*_*p*_ = *L*_*q*_ = *l*/2. In the first proposal, we arrange the AND and CFA plaquettes on two layers and add auxiliary qubits in a middle layer. Inter-gate connections and common input variables are implemented via 3- or 4-body parity constraints imposed on geometrically local qubits. A second proposal shows how additional auxiliary qubits can be used to implement the circuit in a conceptional simpler, two-dimensional layout.

Furthermore, we show how the degeneracy of the ground space can be lifted by adding additional constraints via penalty terms. This isolates (apart from exchanging *p* and *q*) a single ground state, which encodes the information of the prime factors *n* = *p* ⋅ *q*.

#### Three-layer layout

The whole multiplication circuit can be thought of as being composed of individual AND and CFA gates by defining common input variables and by applying one of these operations (building inter-gate connections):

- Identify the sum output of an AND gate with a sum input of a CFA gate (sum-to-sum).
- Connect two CFA gates ’horizontally’, i.e., connect the carry output to the carry input (carry-to-carry).
- Connect two CFA gates ’vertically’ by identifying the sum output of the first with the sum input of the second gate (sum-to-sum).
- Take the carry output of a CFA gate and feed it into the sum input of a second CFA gate (carry-to-sum).

*a. Common inputs:* The binary multiplication circuit [as shown in Fig. 2a] can be rearranged on a 2D grid. This is demonstrated exemplary on a small instance in Supplementary Note 2. Arranged like this, the circuit has common input variables *q*_*j*_, which repeate for all gates in row *j*. Likewise, the variable *p*_*i*_ is a common input for all gates in column *i*. On the parity side, AND and CFA gates are implemented via one or two plaquettes, respectively [see Fig. 3]. Let’s denote the factor-related inputs (not carry or sum inputs) of the gate in row *i* and column *j* with *u*_*ij*_ and *v*_*ij*_. In the parity picture, these inputs are encoded vertically and horizontally within the AND plaquette and within the sum-plaquette of the CFA gate, as depicted in Fig. 3. When these plaquettes are placed on a grid, additional four-body constraints enforce common inputs between these plaquettes. Horizontally introduced four-body constraints impose ${u}_{ij}={u}_{{i}^{{\prime} }j}={q}_{j}$ for all $i,{i}^{{\prime} }$. Likewise, vertical constraints fix all inputs ${v}_{ij}={v}_{i{j}^{{\prime} }}={p}_{i}$ for all $j,{j}^{{\prime} }$. Hence, these plaquettes can be placed in a first layer, where the additional parity constraints implement common input variables [see Fig. 4a and Supplementary Fig. 2b, additional constraints are highlighted in blue].

*b. Inter-gate connections:* Connections of type a-d are implemented via additional parity constraints, as shown in Fig. 4b. The key here is the introduction of one ancilla qubit per unit cell. This extra qubit is enforced to match the value of the carry output ${\sigma }_{{c}^{{\prime} }}=WS$ [see Table 1]. This is imposed by adding an additional 3-body constraint. Type b connections are established between units CFA_*i*,*j*_ and CFA_*i*+1,*j*_ by enforcing

12 $${Z}_{i+1,j}{(SW)}_{i+1,j}{(SW)}_{i,j}={{{{{{{\bf{1}}}}}}}},$$

or equivalently ${\sigma }_{({c}_{i+1,j},{c}_{i+1,j}^{{\prime} })}{\sigma }_{{c}_{i+1,j}^{{\prime} }}{\sigma }_{{c}_{i,j}^{{\prime} }}={{{{{{{\bf{1}}}}}}}}$, which is only possible if ${c}_{i+1,j}={c}_{i,j}^{{\prime} }$. The additional parity connection is shown in Fig. 4b.ii. Connections of type c involve gates CFA_*i*,*j*_, and CFA_*i*−1,*j*+1_ and are imposed by setting

13 $${Y}_{i,j}{(WS)}_{i,j}{X}_{i-1,j+1}{(SW)}_{i-1,j+1}={{{{{{{\bf{1}}}}}}}}.$$

Since $SWY={\sigma }_{{s}^{{\prime} }}$ and *SWX* = *σ*_*s*_ this is only possible if ${s}_{i-1,j+1}={s}_{i,j}^{{\prime} }$. The corresponding parity plaquette is drawn as a red polygon in Fig. 4(b.iii). Cases a and d are implemented similarly and are shown in Fig. 4(b.iv-v).

*c. Programming:* By adding the term ${H}_{{{{{{{{\rm{out}}}}}}}}}^{{{{{{{{\rm{parity}}}}}}}}}(n)$, we penalize all states not resulting in the correct output *n* = *p* ⋅ *q*, i.e., states associated with input numbers *p* and *q*, which multiplied together, do not yield the correct integer *n*. The digits of *n* are available as sum outputs ${s}^{{\prime} }$ to CFA gates, located at the right and lower border of our construction. Since ${\sigma }_{{s}^{{\prime} }}=SWY$, the bit *n*_*k*_ can be encoded into a 2-body interaction between qubit *SW* and qubit *Y* both present as physical qubits. While the first is the ancilla qubit ${\sigma }_{{c}^{{\prime} }}$, the second is part of the carry-plaquette. More specifically, if *n*_*k*_ = 0, the constraint strength should be negative, while if *n*_*k*_ = 1, it should be positive. Moreover, boundary cases could have no previous carry bit, or sum bit, as the rightmost CFA gates. If we still want to use the same parity gates, we have to set these bits to zero. With the identification *σ*_*c*_ = *SWZ*, this is accomplished by imposing a two-body interaction between the ancilla qubit *SW* and qubit *Z*. Figure 4a highlights these interaction terms with a green line between the ancilla qubit and the corresponding qubits from the top layer.

*d. Scaling:* A circuit capable of multiplying *l*/2 times *l*/2 bit number produces an output *n* of size $l=\lfloor {\log }_{2}(n)\rfloor$ bits. Such a circuit consists of *l*/2 AND gates and *l*/2(*l*/2 − 1) CFA gates. Including the carry qubits, there are *l*(9*l* − 10)/4 physical qubits.

If the multiplication device is used to find the factors of an odd bi-prime *n* = *p* ⋅ *q*, both *p* and *q* are odd, i.e., *p*_0_ = *q*_0_ = 1. This makes the first row of AND gates redundant, as AND(*u*, 1) = *u* holds. Consequently, 2*l* qubits related to the AND gates can be removed from our count s.t.

14 $${\#}_{{{{{{{{\rm{phys}}}}}}}}}=\frac{9}{4}l(l-2)$$

indicates the number of physical qubits required within this approach [see Supplementary Figure 1].

#### Planar layout

Although the three-layer construction is more efficient in the number of qubits, it could be challenging to implement. So here we propose a planar layout at the expense of additional ancilla qubits.

For the sake of simplicity, let us first consider an infinite lattice of CFA_*ij*_ gates. Their inputs should be *p*_*i*_, *q*_*j*_, *s*_*i*,*j*_ and *c*_*i*,*j*_, as denoted in Fig. 5(a). Their respective outputs are ${s}_{i,j}^{{\prime} }$ and ${c}_{i,j}^{{\prime} }$ and should be connected to inputs *s*_*i*−1,*j*+1_ and *c*_*i*+1,*j*_.

*a. Common inputs:* As a starting point for the planar layout, we consider the bottom layer of the previous construction. As mentioned, horizontal lines “transmit” the *q*_*j*_ variables, while vertical lines encode and transmit the variables *p*_*i*_.

*b. Inter-gate connections:* We slightly modify the arrangement of sum-plaquettes to make room for embedding the associated carry-plaquettes. As shown in Fig. 5(b), adjacent sum-plaquettes are no longer connected by single plaquettes but by a series of three(four)-body plaquettes. Trivially, a series of square plaquettes would act as a wire to carry the variables *p*_*i*_ and *q*_*j*_. However, to create additional space, squares can be replaced with two consecutive triangles pointing at each other. The carry plaquette (*WXYZ*)_*ij*_ is placed in an elongated shape in the enlarged space. This embedding allows the full-adders logic to be implemented in a (4 × 5)-qubit unit cell equipped with local fields and additional parity constraints. Similarly to a biological cell, this elementary cell has a boundary structure composed of sum plaquettes connected via wires. Not only do these wires carry information in one direction, but they can also act like a membrane that allows information to flow perpendicularly.

The reader might easily check if *PQRS* = **1**, *WXYZ* = **1** holds and all the other parity constraints are full-filled, then the additional qubits have values like *SW*, *SWX*, *SWY,* and *SWZ* as shown in Fig. 5(b). According to Table 1, these spins encode the logical variables ${\sigma }_{{c}^{{\prime} }}$,*σ*_*s*_, ${\sigma }_{{s}^{{\prime} }}$, and *σ*_*c*_. In the presented layout, they are positioned s.t. information is expressed on boundary plaquettes encoded perpendicular to *p*_*i*_ or *q*_*j*_ wires. Thus, two horizontal-adjacent unit-cell directly enforces the carry inter-gate connection. Similarly, diagonally adjacent cells (*i*, *j*) and (*i* − 1, *j* + 1) link their sum variables via an additional information path that leads through the elementary cell (*i*, *j* + 1). This path is represented by the red triangles in Fig. 5(b).

Unlike the infinite lattice of CFAs, the binary multiplier has specific boundary cases. These cases are considered in Supplementary Note 3.

*c. Programming:* The number to be factored is imposed by adding a Hamiltonian ${H}_{{{{{{{{\rm{out}}}}}}}}}^{{{{{{{{\rm{parity}}}}}}}}}(n)$ as a sum of local field terms. These local fields determine the state of the qubits associated with $SWY={\sigma }_{{s}^{{\prime} }}$. For unit cells at the lower boundary of the layout, these qubits are depicted in Fig. 5b. Additionaly, see Supplementary Fig. 4b for a discussion of the right-hand-side boundary cases. Finally, the bottom left unit allows accessing the most significant bit *n*_*l*_ via ${\sigma }_{{c}^{{\prime} }}=SW$.

*d. Scaling:* As depicted in Fig. 5, a single CFA unit can be implemented within the parity framework with 18 qubits arranged on a 4 × 5 array. However, the number of qubits can be improved to 14 per unit cell by considering a planar implementation without restricting to a grid-based layout [see Supplementary Fig. 5]. With this, factoring a number with *l* bits length (when both factors have *l*/2 bits) can be done at the cost of approximately ${\#}_{{{{{{{{\rm{phys2D}}}}}}}}}\approx \frac{7}{2}{l}^{2}$ qubits in a planar layout.

## Discussion

The parity-based Hamiltonians described in the last section can be used to factor numbers *n* of size *l* bits were we assume that both factors fit into a *l*/2-bit register. In general, a sufficient length of the factors is determined by the bounds ${L}_{p}=l:= \lfloor {\log }_{2}(n)\rfloor$ and ${L}_{q}=\lceil \frac{1}{2}(l+1)\rceil -1$^12^. Not knowing the length of the factors in advance is part of the factoring problem and makes it challenging. The extreme cases in which one of the factors is very small or contrary when both are nearly equal in size can be approached classically. Using, e.g., simple trial division, factors up to a certain threshold size of *r* bits could be checked. On the other hand, factoring algorithms such as Fermat’s method performs well if both factors are close in value^5^. When using the RSA protocol, one is interested in making an attack as challenging as possible. Therefore, one can assume that neither of the factors is small nor the same size. To span this range, the circuit must be able to encode the multiplication of (*L*_*p*_ − *r*) bit times *L*_*q*_ bit numbers, resulting in a *l* bit number. Without pre-processing, i.e., *r* = 0, the maximal resources needed are approximately $\frac{3}{2}{\#}_{{{{{{{{\rm{phys}}}}}}}}}(l)$ qubits. This leads to an estimate of 3.4 *l*^2^ qubits in the case of the three-layer proposal and 5.3 *l*^2^ qubits in the planar case [versus 25 *l*^2^ qubits given for a similar approach^32^].

The current state-of-the-art RSA2048 protocol uses a key that is 2048 bits in size. To attack such a problem following our three-layer proposal one would need around 14 million qubits before classical preprocessing.

Alternative approaches for reformulating factorization as an optimization problem are summarized in Supplementary Note 1. They are based on the multiplication table [see Supplementary Table 1] by building factoring equations column by column. Squaring these leads to a QUBO problem involving ${{{{{{{\mathcal{O}}}}}}}}({l}^{2})$ logical variables. Mapping the QUBO problem to annealing hardware introduces another roughly quadratic overhead [see Supplementary Figure 1]. Our problem-specific approach reduces the hardware overhead s.t. we gain a scaling advantage over these approaches in the number of qubits [see also Supplementary Note 7, for a brief discussion on time complexity]. The parity gates and, thus, the whole factorization scheme can be implemented on a variety of platforms: From superconducting qubits like Transmon qubits, KPOs, or arrays of neutral Rydberg atoms^33–37^. See Supplementary Note 8 for a discussion of possible implementations.

Especially in platforms such as superconducting flux qubits, there is a fundamental trade-off between strong coupling and high coherence^38,39^. Here we see a major advantage of our factoring proposal compared to the QUBO approach. The range of interaction strengths in the QUBO model increases with system size and can vary from coupler to coupler. As size increases, these coupling strengths have to be controlled more precisely, challenging the performance of an annealer with a limited dynamic range. In our proposal, the functionality of the parity gates depends on precise local field conditions and parity constraints. Here the local fields only take values in { − 1, 0, 1} and are independent of the instance, i.e., they can be hard-coded into the device. On the other hand, there is no need for precise control over the strength of the parity constraints. Since they act as a penalty, it is sufficient to guarantee that they exceed a threshold, i.e., *k* > 1 [as described in Supplementary Note 4]. The number to be factored is programmed by turning on ${{{{{{{\mathcal{O}}}}}}}}(\log n)$ interactions. There, the magnitude of the interaction is not crucial, but rather its sign. Finally, the fact that information is encoded redundantly allows the parity implementation to provide a simple error detection and mitigation scheme, as discussed in Supplementary Note 5.

## Methods

As our proposal builds on the parity framework, we provide a summary of the methodology behind the parity approach.

### All-to-all connected optimization problems

Connectivity is a significant challenge in mapping optimization problems to hardware, especially when dealing with dense or all-to-all connected graphs. Ref. ^31^ proposed a solution to this problem by introducing a physical variable for each pair of logical variables *σ*_*ij*_ ≔ *σ*_*i*_*σ*_*j*_. These variables are not independent of each other, which allows the original configuration space to be reclaimed in terms of a constrained subspace. Physical variables correspond to spins placed on a 2D grid, and constraints are enforced using penalties in the form of three- and four-body terms that penalize odd numbers of up spins on neighboring spins. The relevant constraints can be constructed from closed loops in the logical graph, e.g., 1 − 3 − 2 − 4. Along such a closed loop, only an even amount of parity changes can occur. In the parity description, four spins, *σ*_13_, *σ*_23_, *σ*_24_, and *σ*_14_, are introduced to keep track of these changes. An even number of parity changes gives rise to constraint

15 $${\sigma }_{13}{\sigma }_{23}{\sigma }_{24}{\sigma }_{14}={{{{{{{\bf{1}}}}}}}}.$$

Introducing penalty terms for sufficiently many independent constraints, an Ising Hamiltonian *H*_Ising_ = ∑_*i*<*j*_*J*_*ij*_*σ*_*i*_*σ*_*j*_ is implemented through the Hamiltonian

16 $${H}_{{{{{{{{\rm{par}}}}}}}}}=\mathop{\sum}\limits_{i < j}{J}_{ij}{\sigma }_{ij}-{c}_{{{{{{{{\rm{penalty}}}}}}}}}\mathop{\sum}\limits_{P\in {{{{{{{\rm{Plaq.}}}}}}}}}\mathop{\prod}\limits_{kl\in P}{\sigma }_{kl},$$

build on programmable local fields *J*_*ij*_*σ*_*ij*_ and problem-independent penalty terms acting on plaquettes of neighboring spins. If the energy penalty *c*_penalty_ is strong enough, it separates the logical subspace from the rest, allowing a one-to-one correspondence with the logical model^40^.

### Higher order parity framework

As a generalization of ref. ^31^ the parity framework introduced in ref. ^29^ can be used to map a *k*-fold product of logical spins onto a single physical parity qubit, e.g., *J*_*ijk*_*σ*_*i*_*σ*_*j*_*σ*_*k*_ → *J*_*ν*_*σ*_*ν*_. This allows the direct encoding of combinatorial optimization problems with arbitrary higher-order *k*-body terms on a square lattice equipped with local fields *J*_*ν*_ and parity constraints. Take, for example, a Hamiltonian including these four terms

17 $${H}_{{{{{{{{\rm{part}}}}}}}}} = \;	{J}_{12}{\sigma }_{1}{\sigma }_{2}+{J}_{45}{\sigma }_{4}{\sigma }_{5}\\ 	+{J}_{123}{\sigma }_{1}{\sigma }_{2}{\sigma }_{3}+{J}_{345}{\sigma }_{3}{\sigma }_{4}{\sigma }_{5}.$$

Introducing four physical variables *σ*_24_ ≔ *σ*_2_*σ*_4_, etc., for each term in Eq. (17) gives rise to the constraint *σ*_12_*σ*_45_*σ*_123_*σ*_345_ = **1**. This follows from

18 $$({\sigma }_{1}{\sigma }_{2})({\sigma }_{4}{\sigma }_{5})({\sigma }_{1}{\sigma }_{2}{\sigma }_{3})({\sigma }_{3}{\sigma }_{4}{\sigma }_{5})={{{{{{{\bf{1}}}}}}}},$$

using ${({\sigma }_{i})}^{2}={{{{{{{\bf{1}}}}}}}}$ and the fact that every logical variable appears twice. The terms specified in Eq. (17) may only represent a subset of terms we wish to implement on hardware. For example, the Hamiltonian could consist of two other terms, *J*_15_*σ*_1_*σ*_5_ and *J*_24_*σ*_2_*σ*_4_, which imply a second parity constraint *σ*_12_*σ*_45_*σ*_15_*σ*_24_ = **1**. Hence, it’s possible to represent this Hamiltonian by six parity variables on two adjacent 4-body plaquettes, each equipped with a 4-body penalty term.

When introducing parity variables, the logical problem is embedded in a higher-dimensional Hilbert space. This embedding requires additional constraints to define the subspace containing the logical problem. A recent study ref. ^29^ investigated how to determine the necessary constraints and a corresponding layout for the physical spins, which they refer to as ’compiling’ the problem for the parity framework. In this context, this work focuses on compiling the factoring problem by first identifying a suitable Hamiltonian for ground-state encoding.

### Supplementary information

Supplementary Information

## Data Availability

Data sharing is not applicable to this article as no datasets were generated or analyzed during the current study.
