# Supplementary material for: Scalable set of reversible parity gates for integer factorization
Source: Commun Phys. 2023 Apr 17;6(1):73. doi: 10.1038/s42005-023-01191-3 (PMC11041783; doi:10.1038/s42005-023-01191-3)
Supplement: Supplementary file 1 — Supplementary Information [file 42005_2023_1191_MOESM1_ESM.pdf]

Supplementary Information: "Scalable Set of Reversible Parity Gates For Integer Factorization"

Martin Lanthaler,[1,*] Benjamin E. Niehoff,[2] and Wolfgang Lechner[1,2]

[1] *Institute for Theoretical Physics, University of Innsbruck, A-6020 Innsbruck, Austria*[†]

[2] *Parity Quantum Computing GmbH, A-6020 Innsbruck, Austria*

I. SUPPLEMENTARY NOTE 1: REVERSE FACTORING AS AN OPTIMIZATION PROBLEM

Most of the combinatorial optimization tasks that arise from real-world problems can be reformulated as QUBO problems, i.e., the problem of finding the minimum of a quadratic objective function

$$Q(x) = \sum_i q_i x_i + \sum_{ij} q_{ij} x_i x_j. \tag{1}$$

$x_i \in \{0, 1\}$ [1]. If one promotes variables x_i to spin operators σ_i ($= \sigma_z$ on spin i) the QUBO problem corresponds to the problem of finding the ground state of an Ising model.

Various algorithms have been proposed to find the global minimum of a given objective function with multiple local minima. An example is Simulated Annealing (SA), in which thermal energy is used to escape local minima and a cooling process leads the system toward low-energy states. Similarly, the meta-heuristic technique known as Quantum Annealing (QA) searches for the global minimum of an objective function by using quantum tunneling in the analysis of the solution candidate space [2–4].

In the broader context of adiabatic quantum computation (AQC) - which is a universal alternative to the gate model [5] - a quantum system prepared in the ground state of the initial Hamiltonian H_{init} is drawn into the ground state of a final Hamiltonian H_{probl} through adiabatic evolution, where H_{probl} itself encodes the optimization problem [4, 6]. For the computation, the time-dependent Hamiltonian varies from H_{init} to H_{probl} via

$$H(t) = (1 - s(t))H_{\text{init}} + s(t)H_{\text{probl}} \tag{2}$$

with $s(t)$ changing from 0 to 1. If this process is performed slowly enough, the quantum adiabatic theorem will ensure the system stays in its instantaneous ground state. In the ideal case, at the end of the sweep, the ground state of H_{probl} is successfully prepared and the optimal solution of the optimization can be obtained by measuring the final state. In the case of QA, the initial Hamiltonian is often referred to as the 'driver' Hamiltonian and is generally of the form $-\sum_i (\sigma_x)_i$, where $(\sigma_x)_i$ denotes the Pauli operator $\sigma_x = |1\rangle\langle 0| + |0\rangle\langle 1|$ acting on qubit i. The ground state of this driver Hamiltonian can be easily prepared as it is the product state $\bigotimes_i |+\rangle_i$ with $|+\rangle = \frac{1}{\sqrt{2}}(|0\rangle + |1\rangle)$ being the plus state. The problem Hamiltonian H_{probl} is usually a 2-local Ising Hamiltonian encoding some QUBO problem Eq. (1).

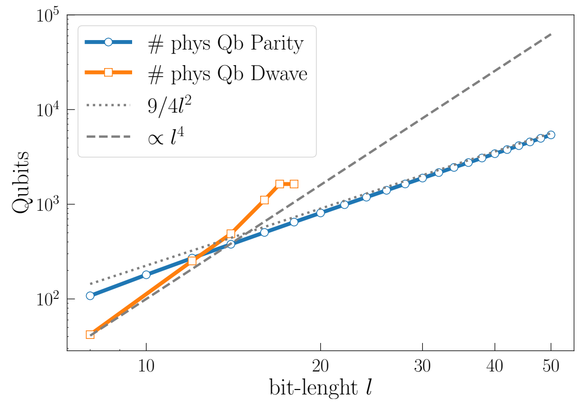

Supplementary Fig. 1. *Comparison of the number of physical qubits.* Our three-layer approach (blue) needs quadratic in $l = \lfloor \log_2(n) \rfloor$ many physical qubits as given by $\frac{9}{4}l(l-2)$. In contrast, the approach using the multiplication table mapping it to a QUBO problem and finally mapping it onto an annealing hardware scale as $\mathcal{O}(\log^4(n))$ (orange) [7]. Experiments ran on a Dwave-2000Q based on the Chimera topology.

Here, we will summarize different approaches to reformulate the factoring problem as an optimization problem. The most frequently examined approaches are based on the binary multiplication table (see Supplementary Tab. I). Within the use of the binary representation, the product $n = p \cdot q$ can be rewritten in terms of their coefficients as

$$n = \sum_{i,j} p_i q_j 2^{i+j} = \sum_k \left(\sum_i p_i q_{k-i} \right) 2^k. \tag{3}$$

However, the coefficients in that expansion cannot be identified with n_k, since $\sum_i p_i q_{k-i}$ can take values ranging from 0 all the way up to $\min(k+1, l-k)$. Collecting the binary products $p_i q_i$ column-wise (see multiplication table Supplementary Tab. I) according to their associated powers 2^k with $k = i + j$, a set of equations can be derived. The complete set of equation, also known as 'factoring equations', includes carry variables like c_{12}. In the particular case of c_{12}, that variable carries the potential overflow, when calculating the sum mod 2 of all terms

* Corresponding author

† Martin.Lanthaler@uibk.ac.at

related to the 2^1 column s.t. $q_0 p_1 + q_1 p_0 = c_{12} \cdot 2 + n_1$ holds. The number of terms each column contains, defines the number of carry variables needed: In the worst case, all of the summands are 1, thus the leading term of the binary expansion of '#(terms)' defines the highest column j such that a carry variable $c_{ij} \neq 0$ is required.

power	2^7	2^6	2^5	2^4	2^3	2^2	2^1	2^0
p					p_3	p_2	p_1	p_0
q					q_3	q_2	q_1	q_0
$1q_0 \cdot p$					q_0p_3	q_0p_2	q_0p_1	q_0p_0
$2q_1 \cdot p$				q_1p_3	q_1p_2	q_1p_1	q_1p_0	
$4q_2 \cdot p$			q_2p_3	q_2p_2	q_2p_1	q_2p_0		
$8q_3 \cdot p$		q_3p_3	q_3p_2	q_3p_1	q_3p_0			
carries	c_{67}	c_{56}	c_{45}	c_{34}	c_{23}	c_{12}		
	c_{57}	c_{46}	c_{35}	c_{24}				
n	n_7	n_6	n_5	n_4	n_3	n_2	n_1	n_0

$$\mathrm{col}_2(p,q,c,n) := q_0p_2 + q_1p_1 + q_2p_0 + c_{12} - (2c_{23} + 4c_{24}) - n_2 = 0$$

$$\min_{p,q,c,n} \sum_i (\mathrm{col}_i)^2 = 0 \Leftrightarrow n = p \cdot q$$

Supplementary Tab. I. *Factoring equations.* Using the binary multiplication table, the factorization problem can be rewritten as an optimization problem. The binary products $p_i q_j$ are arranged column-wise according to their associated power of two. Summing them up along the column $\sum_i p_i q_{k-i}$ and taking into account carry variables factoring equations can be derived. Squaring them defines a cost function having an optimal solution if and only if $n = p \cdot q$.

The factoring equations $\mathrm{col}_i(p,q,c,n)$ define via $\mathrm{cost}(p,q) := \sum_i (\mathrm{col}_i)^2$ a 4-th order polynomial, whose zeros uniquely defines the multiplicative relationship between the variables p_i, q_i, c_{ij} and n_i. By adding ancillary variables, the high-order terms can be substituted by quadratic terms, ensuring that both expressions have the same minimum when restricted to the set of original variables. Finally, the quadratic unconstrained binary optimization problem (QUBO) obtained in this way must be mapped to annealing hardware. This introduces another overhead (in the number of qubits) [7, 8].

Another approach - which we follow in this work - is to introduce carry variables and sum variables for each product $p_i q_j$ appearing in the multiplication table. The carry variables connect different columns of the table, while the sum variables connect different rows and thus divide the entire table into cells. As with the previous method, one must compute the sum over all terms in a given column while balancing carry-out variables associated with higher-order columns. But this time, the sum variables keep track of the partial sum mod 2, while the carry variables just connect adjacent columns. It is common to describe the logic of these individual cells in the language of Boolean circuits. The corresponding cells are described by half-adder (HA) or full-adder (FA) gates [see main text Figure 2(a) for the circuit layout]. Given a previous partial sum 's' from the row above and a carry from the previous column 'c' the relation $s + c + x = 2c' + s'$ defines the new binary sum s' and the new binary carry variable c'. Here, each input x is of the form $p_i q_j$, i.e., can be implemented as the logical AND between the variables p_i and q_j [see main text Figure 2(b)]. In ANF normal form, these relations can be written as $s' = (p_i \wedge q_j) \oplus s \oplus c$ and $c' = (p_i \wedge q_j \wedge s) \oplus (p_i \wedge q_j \wedge c) \oplus (s \wedge c)$, where $\oplus$ denotes the XOR function.

II. SUPPLEMENTARY NOTE 2: A CASE STUDY OF A 3BIT MULTIPLIER

In this section, we give an in-depth discussion of a toy model-sized parity-based multiplier. A small illustrative example of a 3bit × 3bit multiplier is depicted in Supplementary Fig. (2). The input numbers are $p = p_2 p_1 p_0$ and $q = q_2 q_1 q_0$ given in binary expansion. Since p and q are both integers between zero and seven, their product cannot exceed 49. Hence, the output number n fits into a register $n = n_5 n_4 ... n_0$ of six bits length. In order to compute the binary digits of the product integer $n = p \cdot q$, the multiplication circuit presented in the main text Figure 2(a) has to sum up $3^2 = 9$ binary products $p_i q_j$ while respecting carry overflows to higher powers of two. The corresponding circuit is built up with three AND gates and six CFA units as shown in Supplementary Fig. 2(a). As discussed in the main text, each connection between gate nodes has to be compensated with an independent constraint in the physical model. There are two types of such connections: a) Common variable connections, where two input nodes are connected. b) Inter-gate connections, i.e., an output node of a preceding gate is connected to an input node of a subsequent gate.

As illustrated in Supplementary Fig. 2(a, left panel) the variables q_j are simultaneous input to three gates. In that regard, q_0 serves as common input for all three AND gates, while q_1 and q_2 act as inputs for three CFA gates each. In our arrangement the variables q_j repeat 'horizontally'. Likewise, the input variables p_i repeat 'vertically': Each column of gates - consisting of one AND gate and two CFA gates - do all have a common input p_i. Focus on the first row of AND gates. In contrast to the trivial case of three independent and unconnected AND gates, the connections between the input nodes given by q_0, reduce the number of independent variables by two. Since the 3-bit example circuit is implemented over three rows and three columns of gates, there are in total $2 \cdot 2 \cdot 3 = 12$ connections between input nodes.

Supplementary Fig. 2(a, right panel) shows the inter-gate connections of type b). The rightmost AND gates directly output the lowest significant bit n_0 but the other two AND gates feed their outputs s_0 and s_1 into two subsequent CFA gates. Furthermore, the sum output of a CFA gate is connected to a sum input of a second CFA gate twice. On four occasions, two CFA gates are connected from carry output to carry input, and finally, once the carry output is fed into a sum input of an CFA gate. This count gives nine additional connections of type b).

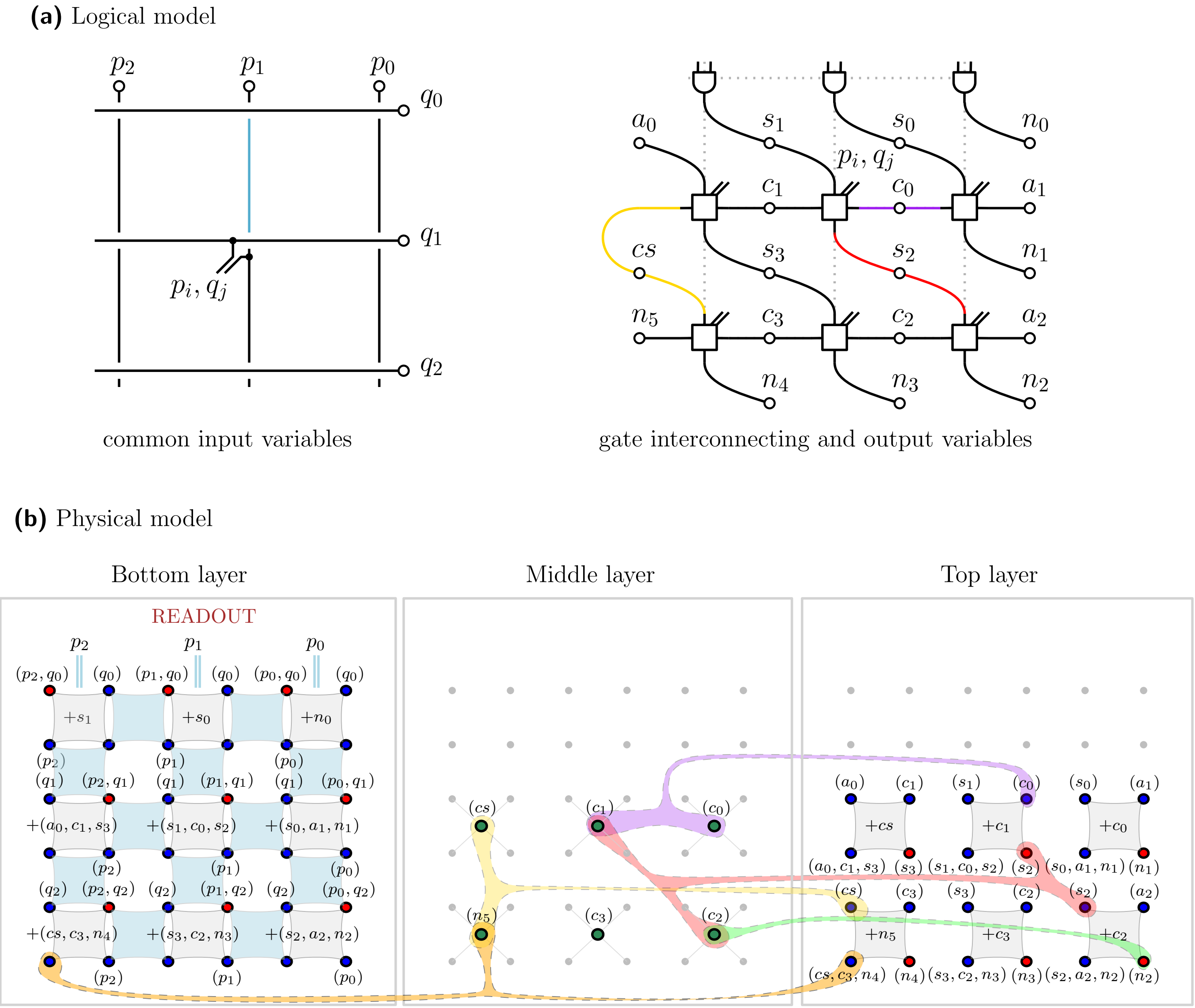

Supplementary Fig. 2. *Labeling of a 3bit × 3bit multiplier.* (a) Logical variables. (b) Labels associated with physical qubits. Here we introduce the shorthand notation $+$(labels). Exemplary, the plaquette in the right-upper corner has a $+n_0$ associated with it. This should be understood that the qubit labels associated with that primary plaquette should all be formally extended with n_0. The obtained labels are (p_0, q_0, n_0), (q_0, n_0),(p_0, n_0) and (n_0). Common input variables relate to blue 4-body terms in the bottom layer (blue). Inter-gate connections translate into 3- and 4-body constraints coupling qubits from different layers (yellow, orange, red, and violet). Two-body constraints are introduced to program the device (green).

Summarizing, $12 + 9 = 21$, connections suffice to build the 3bit × 3bit multiplier from basic AND and CFA gates. Supplementary Fig. 2(a) shows the labeling of the necessary 24 logical variables. Six of them store the input p, q, and six hold the output information n. Furthermore, we need four sum variables s_0, s_1, s_2, s_3 and four carry variables c_0, c_1, c_3, c_4 and a special variable cs for connecting the leftmost carry output to the sum input of the next row. Finally, since we make repetitive use of the CFA gate - even when there is no previous carry or sum - we introduce three auxiliary variables a_0, a_1, a_2. Setting these inputs to zero enables the implementation of the required half-adders within the implementation of a full adder.

The translation into the parity model proceeds as described in the main text: Each AND gate is implemented as a plaquette of 4 qubits while CFA gates are realized within two plaquettes of 8 qubits in total. Furthermore, gate-node connections are translated into parity constraints connecting bare plaquettes. Main text Figure 4(b) shows the basic translation steps.

Let us focus for a moment on connections of type a). Figure 4(b.i) and Fig. 4(b.ii) show the cases of horizontally repeating q_j input variables. As we have discussed

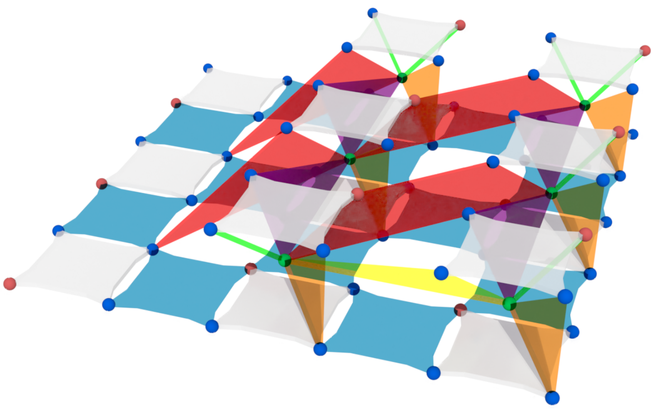

Supplementary Fig. 3. *Three-dimensional visualisation of the 3bit × 3bit factorization device.* In contrast to Fig. 4(b) from the main text, all parity plaquettes are shown and color-coded according to their purpose. Qubits are represented by small spheres where the color encodes the local field strength. White plaquettes denote primary plaquettes, while blue ones are connected to common input variables. Yellow, orange, red, and violet plaquettes represent inter-gate connections while green two-body interactions program the device.

in the main text, the common input variable q_j appearing in neighboring gates translates to an additional 4-body constraint in the parity model connecting both sum plaquettes. Besides horizontal connections of input variables, we also have vertical repeating variables p_i. Similar to the horizontal case, these connections lead to an additional 4-body constraint connecting the sum plaquettes. To see this it is insightful to have a look at a simpler circuit: Consider a 2D-grid of AND gates of size $k \times k$. We connect the first input nodes of the gates column-wise and the second input nodes row-wise, i.e., the circuit has $2k(k-1)$ connections between input nodes. We translate the logic of the AND gates into the parity model using k^2 plaquettes. We enumerate the AND gates by $[i, j]$, where i denotes the column index and j the row index [as in Supplementary Fig. 2(a, left panel)]. If we call $s_{i,j}$ the sum output of the $[i, j]$-AND gate, the corresponding plaquette has labels $(s_{i,j}), (p_i, s_{i,j}), (q_j, s_{i,j})$ and $(p_i, q_j, s_{i,j})$. We can group these into sets $R := \{(s_{i,j}), (q_j, s_{i,j})\}$ and $L := \{(p_i, s_{i,j}), (p_i, q_j, s_{i,j})\}$ or alternative into $D := \{(s_{i,j}), (p_i, s_{i,j})\}$ and $U := \{(q_j, s_{i,j}), (p_i, q_j, s_{i,j})\}$. The two tuples from the set 'right' R and 'left' L formally differ by q_j - independent of the column i, while the elements in 'down' D and 'up' U differ by p_i for all row indices j. Neighbouring AND parity plaquettes $[i, j]$, $[i+1, j]$ can thus be connected with a parity constraint involving qubits labeled by, e.g., $L_1 := \{(p_i, s_{i,j}), (p_i, q_j, s_{i,j})\}$ and $R_2 := \{(s_{i+1,j}), (q_j, s_{i+1,j})\}$, i.e., the left set of the first plaquette and the right set of the second plaquette. Similarly, vertically neighboring AND parity plaquettes $[i, j]$, $[i, j+1]$ can be connected with a parity constraint on qubits with labels in $D_1 := \{(s_{i,j}), (p_i, s_{i,j})\}$ and $U_2 := \{(q_{j+1}, s_{i,j+1}), (p_i, q_{j+1}, s_{i,j+1})\}$. The crucial point is, by carefully arranging the labeling in between the plaquettes, horizontal and vertical parity constraints are simultaneously available. A possible way is to arrange the labeling in each plaquette such that the upper qubits are labeled with labels from the set U and respective the lower, the right, and the left qubits. In that sense, the qubit in the right-down corner should get the label $RD = R \cap D = (s_{i,j})$. Analog we get $LD = (p_i, s_{i,j})$, $RU = (q_j, s_{i,j})$ and $LU = (p_i, q_j, s_{i,j})$. It is straightforward to check this arrangement yields $2k(k-1)$ new 4-body parity constraints.

With this analysis in mind, we focus again on the arrangement of AND and CFA plaquettes found in the multiplier circuit. We have already emphasized in the main text: One of the two CFA plaquettes is conceptually similar to the AND parity plaquette, i.e., the corresponding labeling is obtained by formally replacing the sum output label s by the combination s, c, s'. Besides this difference, the overall structure of the sum plaquettes is the same as the 2D-grid example of AND gates. Again, the input variables p_i repeat vertically and the variables q_j horizontally. With this, it is easy to understand that the AND plaquettes and the sum plaquettes can be arranged in a 2D layer with $2k(k-1)$ new 4-parity constraints acting on 2×2 plaquettes. In the case of $k = 3$ there are 12 new constraints as depicted in Supplementary Fig. 2(b, left panel). These plaquettes are arranged in the first layer. The drawing in Supplementary Fig. 2(b, left panel) shows the labeling of the physical qubits. Since several indices appear repeatedly amongst qubits belonging to the same plaquette we introduce a shorthand notation: We formally split the string of labels into a common part and unique parts. The common part +(common-labels) is presented in the center of the plaquettes and the unique and distinct parts are represented as labels associated with the qubits. Whenever the reader finds +(common-labels) on the center of a plaquette, this should be interpreted s.t. each short-hand labeling of the four involved qubits should be extended by the common part (common-labels) to form the actual labeling string. For example, the labeling of the plaquette in the upper-right corner should read as $(p_0, q_0, \mathbf{n_0})$, $(q_0, \mathbf{n_0})$, $(p_0, \mathbf{n_0})$ and $(\mathbf{n_0})$ and the common part is $\mathbf{n_0}$.

So far we discussed the AND and sum plaquettes which can be arranged into a first layer. Additionally, there are six plaquettes - related to the six CFA gates (the carry plaquettes) - still disconnected from the first layer. Connection of type b), i.e., inter-gate connections, translate into parity constraints, fuse the carry plaquettes to the first layer. The basic construction steps are described in the main text and are depicted in Fig.4(b). There we described, that for each pair of sum and carry plaquettes, a 3-body parity constraint, and a carry qubit are introduced s.t. the constraint fixes the value of the corresponding carry qubit. While the carry plaquettes can be arranged on a second layer - above their sum counterparts [see Supplementary Fig. 2(b, right panel)] - the

carry qubits can be arranged in-between these layers in a middle layer [see Supplementary Fig. 2(b, middle panel)]. With the help of the six $c_0, c_1, cs, c_2, c_3, n_5$ auxiliary carry qubits, the missing nine constraints associated to connections of type b) can be constructed [See Tab. II].

comm.var.	constraint	# qubits
s_0	$(s_0), (s_0, c_0), (c_0)$	3-body
s_1	$(s_1), (s_1, c_1), (c_1)$	3-body
s_2	$(s_2, c_1), (c_1), (s_2, c_2), (c_2)$	4-body
s_3	$(s_3, cs), (cs), (s_3, c_3), (c_3)$	4-body
c_0	$(c_0), (c_0, c_1), (c_1)$	3-body
c_1	$(c_1), (c_1, cs), (cs)$	3-body
c_2	$(c_2), (c_2, c_3), (c_3)$	3-body
c_3	$(c_3), (c_3, n_5), (n_5)$	3-body
cs	$(cs), (cs, n_5), (n_5)$	3-body

Supplementary Tab. II. *Common variables and associated constraints.* List of constraints which are related to inter-gate connections for the 3bit example.

Here, labels with two variables refer to qubits in the top layer, and the others containing a single variable are either associated with the auxiliary qubits in the middle layer or the output qubit of the first row of plaquettes in the lowest layer [see Supplementary Fig. 2]. The column 'comm.var.' shows the common variable associated with inter-gate connections, i.e., connections of type b). Each such inter-gate connection in the circuit allows the construction of a parity constraint in the physical model. Some of them are highlighted in Supplementary Fig. 2(b), showing a 4-body constraint in red and 3-body constraints in violet or yellow. See also Supplementary Fig. 3 for a 3D visualisation of the 3bit × 3bit multiplier example. In total 12+9 = 21 parity constraints compensate the 21 identifications made by common input connections and inter-gate connections in the process of building the multiplication circuit from basic gates. The 3bit × 3bit multiplying parity design can be programmed by introducing local fields on qubits associated with the labels n_0 and n_5 and 2-body terms related to the sets of labels $\{(c_2), (n_2, c_2)\}$, $\{(c_3), (n_3, c_3)\}$ and $\{(c_5), (n_4, c_5)\}$. Supplementary Fig. 2(b) shows one of the two body interactions necessary to program the bit n_2 in green. Additionally, some carry inputs a_1, a_2 and a sum input a_0 have to be set to zero by adding interactions on $\{(cs), (a_0, cs)\}$, $\{(c_0), (a_1, c_0)\}$ and $\{(c_2), (a_2, c_2)\}$. This allows mimicking the logic of a half-adder within the implementation of CFA gates.

III. SUPPLEMENTARY NOTE 3: PLANAR LAYOUT

As mentioned in the main text, the multiplication circuit can be built, w.l.o.g., from CFA gates and hence, with their parity-equivalent introduced in Fig. 5(b). However, boundary cases must be treated somewhat differently. On the right boundary, the carry input has to be set to zero, while the leftmost gates have to be connected via their sum output to the sum input of the leftmost gate in the next row. This link from cell (i, j) to $(i, j+1)$ could be easily implemented using the unit cells introduced in Fig. 5 by connecting the carry output with a series of triangular plaquettes to the sum input of the unit below. However, more efficiently, this information channel can be integrated into the unit-cell (i, j) as depicted in Supplementary Fig. 4(a). Likewise, panel (b) of Supplementary Fig. 4 shows how the right border can be simplified, while panel (c) shows how the first row of AND gates can be included.

The proposal so far is strictly connected to a two-dimensional arrangement on a grid, where qubits are placed on the intersection points of the grid, while (three or four-body) constraints are only allowed between neighboring qubits on the 2×2 plaquettes of the grid. However, if we lift this restriction, the CFA implementation presented in Fig. 5 is not optimal in terms of qubit numbers. In this regard, Supplementary Fig. 5 shows a layout that is not strictly based on the 2D grid but is still planar (in the sense it's 2D and has no "overlapping" constraints). Using this layout allows the CFA to be implemented with 14 qubits per unit cell.

IV. SUPPLEMENTARY NOTE 4: CONSTRAINT STRENGTHS

In the parity framework, the logic of the multiplication circuit is implemented via local fields and constraints on 3- or 4-body plaquettes as described in the main text in Sec. "Parity mapping". These constraints ensure that the ground space of the spin model faithfully implements the logic of the multiplication circuit. The penalties for non-logical states should be neither too low nor too high: While too low penalties do not allow (energetic) isolation of the logical ground states, too high constraints can be disadvantageous as they suppress transitions during different excitation sectors [9] [see also the discussion in Supplementary Note 7].

Here, we will discuss the three-layer construction first. However, the results immediately apply to the planar case. To determine a lower bound on the parity constraint strengths, we discuss each component of the circuit, starting with the AND gate.

The parity-gate Hamiltonian defined in main text by Eq. (9) consist of the local field term $H_{\mathsf{AND}}^{\text{local fields}}$ and the penalty term $H_{\mathsf{AND}}^{\text{penalty}}(k)$. If $k = 0$, all the states belonging to the logical subspace do have an energy of -2. However, the state having all spins aligned with the local fields, i.e., $|1000\rangle_{\text{phys}}$ does have a lower energy of -4. Fortunately, this state can be distinguished from the logical states via the 4-body penalty term $H_{\mathsf{AND}}^{\text{penalty}}(k)$. In order to establish the one-to-one correspondence mentioned in Table (III) it is sufficient to split the even subspace from

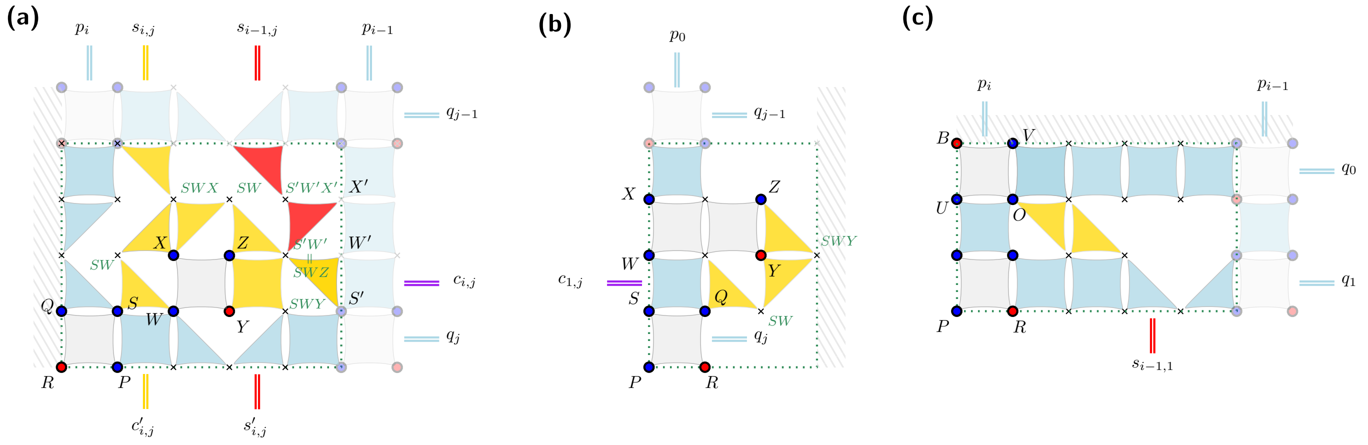

Supplementary Fig. 4. *Boundary cases for the planar layout.* (a) Left boundary: The carry output of gate $\mathsf{CFA}_{i,j}$ is connected to the sum input of gate $\mathsf{CFA}_{i,j+1}$. (b) Full-adder gates on the right boundary have no carry input $c_{0,j} = 0$. (c) First row of $\mathsf{AND}_{i,0}$ gates connect their sum output via unit $(i, 1)$ to input $s_{i-1,1}$.

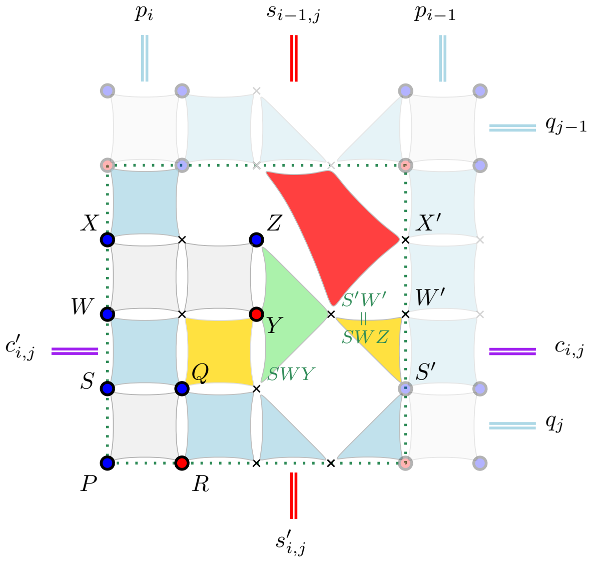

Supplementary Fig. 5. *Smallest planar implementation found.* In contrast to the construction presented in main text Fig. 5(b) the CFA unit can be implemented with at most 14 qubits if parity constraint are not restricted to 2×2 plaquettes.

logical state $\lvert u, v, s = \mathsf{AND}(u, v)\rangle$	parity-encoded state $\lvert (u, v, s), (v, s), (u, s), (s)\rangle$
$\lvert 000\rangle$	$\lvert 0000\rangle$
$\lvert 010\rangle$	$\lvert 1100\rangle$
$\lvert 100\rangle$	$\lvert 1010\rangle$
$\lvert 111\rangle$	$\lvert 1001\rangle$

Supplementary Tab. III. *Logical subspace of the AND gate.* The table lists the one-to-one correspondence between ground state manifold of H_{AND} and the ground space of the associated $H^{\text{parity}}_{\mathsf{AND}}$ Hamiltonian. States not on this list, which differ from the left column in the last bit s are mapped onto the corresponding physical state where all four spins are inverted, e.g., $\lvert 001\rangle \mapsto \lvert 1111\rangle$.

the odd one with an energy difference of $2k$. For $k > 1$ the state $\lvert 1000\rangle_{\text{phys}}$ has an energy larger than any of the states listened in Tab. (III). Furthermore, an increased strength $k > 3$ opens a gap between the manifolds of all odd and even states.

The parity CFA gate, defined in the main text by Eq. (10), consists of two distinct plaquettes, each of four spins [see main text Fig. 3]. Like the parity AND gate, both plaquettes are equipped with local fields, with strengths $+1$ appearing once and -1 three times. Hence, the same argument as before shows $k > 1$ is a sufficient choice for the energy penalty.

In the process of assembling individual parity gates to emulate the multiplication circuit, additional three- and four-body constraints must be introduced. Common input variables are compensated via 4-body plaquettes [see Supplementary Fig. 2(b) blue plaquettes]. It turns out that each of these 4-body plaquettes contains a spin with a local $+1$ field, while the remaining three have a -1 field. This is again the same situation as with the AND plaquette. If one considers only the primary AND and CFA plaquettes and those for the common input variables, the ground state space of the physical model is highly degenerate. For each such ground state, each individual 4-body plaquette contributes energy of $-2 - k$. This degeneracy is lifted by implementing the inter-gate connections using additional 3- and 4-body constraints. Here, a constraint strength $k > 0$ distinguishes between states of correct inter-gate connectivity and false ones. Finally, Sec. c."Programming" shows how we can single out one specific ground state by introducing a 2-local term $H^{\text{parity}}_{\text{out}}(n)$. The corresponding 2-body terms have the form $\pm k \cdot \sigma_{s'} \sigma_{(n_i, s')}$, where the sign is defined the i'th bit of n. A penalty strength $k > 0$ for all of the 2-body terms appearing in $H^{\text{parity}}_{\text{out}}(n)$ opens up a gap of at least

$2k$ between states leading to the correct product n and others leading to $n' \neq n$. It can be convenient to set all constraint strengths to the same value, e.g., $k = 2$ should be an appropriate choice.

V. SUPPLEMENTARY NOTE 5: BACK-TRANSLATION/ERROR MITIGATION

After successful preparation of the final Hamiltonian ground state by any heuristic, e.g., by quantum annealing, the parity-encoded information must be translated back into logical variables. To do this, the physical qubits are measured in the computational basis, which provides a list of assignments

$$\sigma_{\omega_1} = \delta_1, \sigma_{\omega_2} = \delta_2, \ldots, \sigma_{\omega_r} = \delta_r, \tag{4}$$

with $r = \#_{\text{phys}}$, labels $\omega_i \in \{1, ..., \#_{\text{logi}}\}^r$ and $\delta_i \in \{-1, 1\}$. The back translation problem is to determine the values of the logical spins σ_i, where the result of the physical spins is δ_i and their connection is given by the parity translation Eq. (7) in the main text.

First, note that multiplying elements from $\{-1, 1\}$ is isomorphic to the XOR operation (or addition modulo 2) on variables $\{0, 1\}$. Thus, by changing the variables $s_k = (1 - \sigma_i)/2$ and $d_k = (1 - \delta_i)/2$, Eq. (4) corresponds to the system

$$\begin{aligned} s_{\omega_{11}} \oplus s_{\omega_{12}} \oplus ... \oplus s_{\omega_{1|\omega_1|}} &= d_1 \\ s_{\omega_{21}} \oplus s_{\omega_{22}} \oplus ... \oplus s_{\omega_{2|\omega_2|}} &= d_2 \\ &\vdots \\ s_{\omega_{r1}} \oplus s_{\omega_{r2}} \oplus ... \oplus s_{\omega_{r|\omega_r|}} &= d_r \end{aligned} . \tag{5}$$

Since $x_1 \oplus ... \oplus x_m = y \Leftrightarrow x_1 \oplus ... \oplus x_m \oplus y = 0$ the system given by Eq. (5) is equivalent the SAT formula

$$\left[\neg s_{\omega_{11}} \oplus ... \oplus s_{\omega_{1|\omega_1|}} \oplus d_1\right] ... \left[\neg s_{\omega_{r1}} \oplus ... \oplus s_{\omega_{r|\omega_r|}} \oplus d_r\right], \tag{6}$$

i.e., to the problem of finding a satisfying assignment of the variables s_i. According to Schaefer's dichotomy theorem [10] the problem $\mathsf{XOR\text{-}SAT}$ lies in P and can be solved by Gaussian elimination (more precisely, Eq. (5) is equivalent to a $\mathsf{LinEqmod2}$ problem). Since the number of logical variables scales quadratically with respect to the size of our problem $l = \lfloor \log_2(n) \rfloor$, the back translation can be performed efficiently.

This efficient back-translation applies to any parity-encoded problem in general. Here, we are satisfied to obtain/read out the factors p and q. Calculating one of them is enough to efficiently calculate all other logical variables.

Let us focus again on the three-layer construction introduced in the main text Sec. "Three-layer layout". Here, the information about the factors p and q is encoded in a redundant way. To see this, take a physical state that obeys all parity constraints. As the labeling layout depicted in Supplementary Fig. 2(b, left panel) shows, we can extract all the bits of p from the first row of qubits. However, the same applies to the 3rd and 5th row of physical qubits. Under the promise, we can prepare the true ground state of the final Hamiltonian, only $\mathcal{O}(\log n)$ physical qubits need to be read out to learn the prime factors.

Having a low energy state we can measure all the physical qubits. Using the additional information, we can hope to mitigate some errors introduced by the state preparation task. A simple strategy could be:

- Read out all qubits from the bottom layer
- For every other row r, decode the data to get a candidate factor p'. Do the same column by column to get candidates q'.
- Check for each candidate factor p' and q' whether it is actually a factor of n.

Note, that this procedure returns $\mathcal{O}(\log n)$ candidate factors, and the last step can be performed efficiently. Supplementary Fig. 6(inset) shows the improvement in the probability of success when this decoding scheme is applied to a toy model simulation of factoring $n = 6$. We counted the decoding as a success if one of the candidates provided a factor. In this particular case, shown in Supplementary Fig. 6, the state space of logical variables is very small, s.t. even short annealing times give good performance. This is because a randomly chosen physical state can be decoded to one of the factors 2 or 3 by the strategy with probability 3/4.

We admit, this is a fairly simple scheme and that more sophisticated error correction techniques could be much more promising. Mainly because the parity constraints can be interpreted in the context of stabilizer codes. Single spin flips on physical qubits can be detected by violating adjacent parity constraints. By construction, logical spin-flips are constraint-invariant in the parity model, i.e., they do not leave the subspace of states that satisfy all parity constraints. This is due to the fact that a logical spin-flip translates into a physical model excitation in which locally adjacent spins are flipped s.t. an even number of spins per parity plaquette are involved. Nevertheless, they are locally detectable by their induced pattern on primary plaquettes on adjacent parity gates. Easiest to see for the AND parity gate [see Supplementary Tab. III], where four out of the 16 states make up the logical ground space. There are 4 other states, with the energy of +4, w.r.t., the logical ground space, which satisfy the parity constraint but do not encode a valid codeword connected to the logic of an AND gate. By flipping either all four spins or two of them, we can reach a state that still satisfies the parity condition, but the logical ground space is left for one of the states $|1111\rangle, |0011\rangle, |0101\rangle$ or $|0110\rangle$. Up to relabeling of the spins, these patterns serve as a fingerprint for detecting a logical spin flip observable on primary plaquettes.

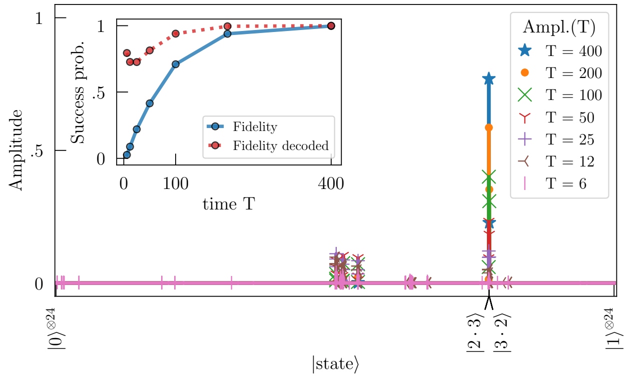

Supplementary Fig. 6. *Annealing simulations for $n = 6$.* The main figure shows the probability distribution over all basis states for different annealing times T (in units of $[\hbar H^{-1}]$). The inset shows the success probability in dependence on the annealing time T, before and after applying a simple decoding strategy. Note that both states $|p \cdot q\rangle = |2 \cdot 3\rangle$ and $|3 \cdot 2\rangle$ are a valid factorisation of 6. All constraint strengths are set to $k = 2$.

This shows that we can detect and correct physical and logical spin flips, in the case they are not too likely to occur. Conversely, there are many other (energetic) low-lying states that are unlikely to allow for a good error correction scheme. For example, consider a state where all but one of the parity constraints are satisfied. Let this constraint be associated with either a carry overflow or a sum connection between gates, i.e., either the purple 3-body constraint or the red 4-body constraint as depicted in the main text Fig. 4(b). This invalid constraint would implement a circuit where we accidentally invert an intermediate carry or sum variable. Instead of the number n we tried to factor the number $n' = n \pm 2^k$ for some k. If n' allows factorization (is not prime and fits in our register), the energy of this state is $+k$ above the ground state of $H^{\text{parity}}_{\text{factor}}(n)$. In such a case, we believe the best strategy is to discard the result and sample a new state.

VI. SUPPLEMENTARY NOTE 6: SIMULATIONS

In this section, we present simulation results for a toy-model-sized implementation of our proposed parity-factorization scheme. We're quite limited in terms of the number of qubits, so here we'll focus on the smallest and simplest instance, where both factors $p = p_1 p_0$ and $q = q_1 q_0$ are 2-bit numbers. Moreover, we choose the three-layer construction to have as least overhead as possible. Valid products that can be formed from two 2-bit numbers are $N = (0, 1, 2, 3, 4, 6, 9)$. In general, multiplication as a group action is associative, i.e., the number $n = 6$ can also be written as $6 = 2 \cdot 3$ and $6 = 3 \cdot 2$. Although, in our physical implementation both factorizations are represented by different states. If $n = 6$, our Hamiltonian has a degenerate ground space spanned by states $|p, q\rangle = |2 \cdot 3\rangle$ and $|3 \cdot 2\rangle$. Also, zero acts as a neutral element s.t. there are seven different ways to factor zero. Given a number $n \in N$ to be factored $d_N = (7, 1, 1, 2, 1, 2, 1)$ indicates the number of possible factorizations and thus also the dimension of the ground space. Altogether there are 16 valid triples $(p, q, n = p \cdot q)$ and thus our subspace of valid multiplications is spanned by 16 different states.

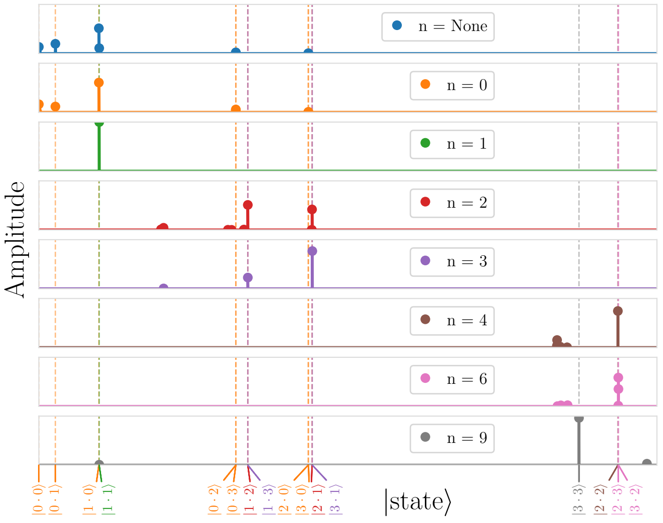

Supplementary Fig. 7. *Annealing simulations for all possible outputs numbers n.* The first panel includes the case where no number is specified. Constraints strengths of $k = 2$ are used and annealing times are set to $T = 200$ (in units of $[\hbar H^{-1}]$). For better representation, we rescale the x-axis compared to Supplementary Fig. 6 by $x \mapsto \sqrt[4]{x+1} - 1$.

The classical 2bit $\times$ 2bit circuit can be constructed from two AND gates and two CFA gates. Compare Supplementary Fig. 2(a) where the corresponding 3-bit circuit is shown and imagine that the most significant row q_2 and column p_2 are missing. There are 8 input and output variables $p_0, p_1, q_0, q_1, n_0, n_1, n_2, n_3$, a sum and a carry variable s_0 and c_0 and two auxiliary variables a_0, a_1, both set to zero. The physical parity model consists of two AND plaquettes of 4 qubits each and two CFA parity gates of 9 qubits each [compare again the 3-bit example in Supplementary Fig. 2(b)]. In total, this makes 26 physical qubits.

We aim to find one of these states using a linear quantum annealing schedule as presented in Supplementary Note 1. Since it is already challenging to simulate quantum annealing on 26 qubits we use a simple trick to reduce the number of physical qubits to 24. For this, we eliminate a physical qubit for each parity CFA gate. This is possible because in the 2bit $\times$ 2bit case both full adders either have no previous carry $a_1 = 0$ or no previous sum $a_0 = 0$. In the first case, the carry qubit c_0 is always aligned with the qubit labeled (a_1, c_0), which is one from the carry plaquette. In the second case, the carry-out (which in the case of the 2bit example is the bit n_4) has

always to be aligned with the qubit labeled (a_0, n_4). In either case, we eliminate one of these qubits by concatenating them, i.e., using a single qubit while all constraints and local fields affecting either qubit must be present afterward.

Supplementary Fig. 6 shows the result when we try to find one of the correct states $|2{\cdot}3\rangle$ or $|3{\cdot}2\rangle$, i.e., the factors of $n = 6$. All constraint strengths are set to $k = 2$. Here we have simulated the full unitary evolution starting from the ground state $|\psi_0\rangle = |+\rangle^{\otimes 24}$ with varying annealing times T using a linear scheme $s(t) = t/T$. The resulting state can be formally written as

$$|\psi(t)\rangle = \mathrm{Texp}\left[-\frac{i}{\hbar}\int_0^t H(\tau)\mathrm{d}\tau\right]|\psi_0\rangle, \qquad (7)$$

where $H(t)$ is the interpolating Hamiltonian Eq. (2). The resulting probability distributions over the entire state space of approximately 17 million (classical) states are shown. We conclude, if the annealing rate is too high (too low total annealing time T), we encounter diabatic excitations, and a large number of states are populated. The performance increases with a slower speed and it is more likely that one of the correct states can be found by the protocol. This behavior is shown in the inset, using the fidelity $\mathcal{F}_{n=6} = |\langle 2\cdot 3|\psi(T)\rangle|^2 + |\langle 3\cdot 2|\psi(T)\rangle|^2$ as a measure of success. Furthermore, we showcase the result of applying a simple decoding strategy as described in Supplementary Note 5.

We examined all possible 2bit × 2bit products $n \in N$ in Supplementary Fig. 7 for the same annealing time $T = 200\,[\hbar H^{-1}]$. Additionally, we annealed into the subspace of all 16 states that describe a valid multiplication. In cases with a degenerate solution space, we observe the well-known fact that not all ground states are found with the same probability [11]. However, in all cases at least one of the possible factorizations can be found with high probability.

VII. SUPPLEMENTARY NOTE 7: PROSPECTS ON TIME COMPLEXITY

The main goal of this work is to design a realistic and programmable device in the form of a local spin Hamiltonian whose ground state encodes the unknown factors of a given number two n. But eventually, that ground state needs to be prepared, and there are a variety of quantum optimization heuristics to choose from. From "pure" quantum algorithms such as quantum annealing to a variety of hybrid, classical-quantum approaches. One of the major bottlenecks in quantum annealing is the existence of a quantum phase transition during time evolution. Several attempts have been made to mitigate the problem of first-order phase transitions, ranging from non-stoquastic Hamiltonians [12], inhomogeneous driving of the transverse field [13, 14] or diabetic quantum annealing [15, 16]. In contrast, hybrid algorithms can better handle erroneous small-scale quantum devices. The Quantum Approximation Optimization Algorithm (QAOA) was proposed alongside the Variational Quantum Eigensolver (VQE) [17, 18] as one of the most important hybrid classical-quantum algorithms [19].

Here we focus on an annealing approach as described in Supplementary Note 1. There, the ground state is prepared by a slowly changing process where at each time step the system is in its instantaneous ground state, starting from an easy-to-prepare ground state. The exact nature of this slow variation is quantified in terms of the adiabatic theorem, which exists in many different variants, varying in assumptions and rigor. The "popular" adiabatic condition states that the total evolution time T should be large on the time scale defined by the square of the inverse gap. A slightly more rigorous estimate is given if Ref. [20] by

$$T \sim \mathcal{O}(\max_s \|\dot{H}(s)\|/\Delta^2), \qquad (8)$$

where Δ denotes the minimum gap $\Delta = \min_s \Delta(s)$. If the schedule is linear, then $\dot{H}(s) = -H_0 + H_1$ is constant in s. This quantity is bounded by $\|\dot{H}(s)\| \leq \|H_0\| + \|H_1\|$, i.e., given by the norm of the initial and the final Hamiltonian. While it is notoriously difficult to compute or to bound the minimum gap, the norms $\|H_0\|$, $\|H_1\|$ are more accessible. More precise, $\|H_0\|$ is diagonal in the x-base, while $\|H_1\|$ is a classic Hamilton operator. It's easy to see that $\|H_0\| = 2\#(\text{phys.qubits})$.

With these considerations, we argue that a small spectral radius (which is equivalent to the operator norm H since for a Hermitian operator $\|H\| = \max_{\lambda\in\sigma(H)} \lambda$) of the final Hamiltonian is desirable. We will show that the scaling properties of these parameters differ significantly when we compare our parity gate approach with the standard multiplication table approach. Our main argument boils down to the observation that all parity gates have local fields of $\{-1, 0, 1\}$ while the strengths of the parity constraint can be chosen to be the same regardless of the size of the number to factor, i.e., they are of order $\mathcal{O}(1)$ as shown in Sect. IV.

The multiplication circuit is built from $\mathcal{O}(\log^2 n)$ gates implemented by the same number of parity gates. Each gate is constructed from a constant number of physical qubits and includes a constant number of plaquettes. A hypothetical state that violates all local fields and all parity constraints simultaneously would have an energy of order $\mathcal{O}(\log^2 n)$. Which shows that $\|H_1\|$ is at most $\mathcal{O}(\log^2 n)$. In particular, $\|H_1\| \leq 5l^2 - 8l$ with $l = \log_2(n)$ as shown in Supplementary Fig. 8.

On the other hand, as pointed out by [8], the range of QUBO coefficients scales as $\mathcal{O}(\log^2 n)$. To see this, the reader can take a look at Tab. I, which presents a simple column-based method to define an optimization version of the factorization problem. The binary products $p_i q_j$ must be added up column by column, taking into account the carry variable c_{ij}. Let's assume for a moment that n has l bits, while both factors have $k = l/2$ bits. Focus on the kth column. The corresponding factoring equation

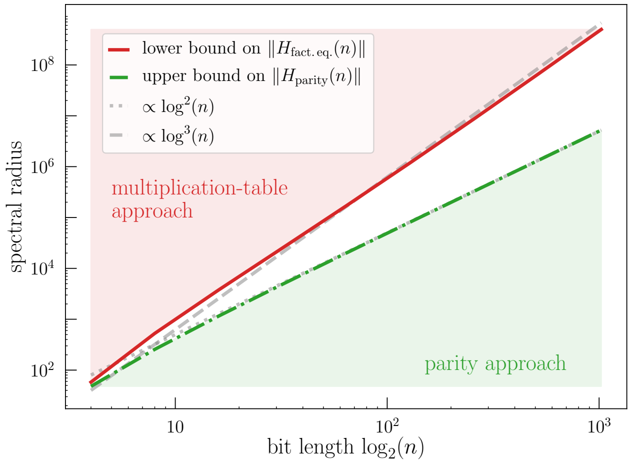

Supplementary Fig. 8. Spectral radius $\|H_1\|$ in case of the parity implementation and a convenient multiplication-table approach.

read as follows

$$\sum_{i=0}^{k} p_i q_{k-i} + c_{\text{in}} - c_{\text{out}} - n_k = 0. \tag{9}$$

Here c_{in} denotes terms that take into account previous carries (coming from lower power-of-2 columns) and c_{out} are terms containing carry variables connecting the column k to subsequent columns of higher powers. In general, c_{out} is of the form

$$c_{\text{out}} = 2c_{k,k+1} + 4c_{k,k+2} + \cdots + 2^m c_{k,k+m}. \tag{10}$$

Here we can think of $(c_{k,k+m} \ldots, c_{k,k+1}, n_k)$ as a $(m+1)$-bit register that should be big enough to hold the value$\sum_i p_i q_{k-1} + c_{\text{in}}$. The sum contains k terms, and in the extreme case, if all are one, results in a sum of k. Even without preceding carries c_{in} we can conclude that $m \sim \log k \sim \log l$ s.t. the highest coefficient 2^m is of the order $\mathcal{O}(l) = \mathcal{O}(\log n)$. In order to translate the factoring equation into a cost function with unique optima, the equations must be squared. Consequently, the coefficients must also be squared. To transform this cost function into a QUBO of order $\mathcal{O}(\log^2 n)$, auxiliary variables are introduced. This converts each of the higher-order terms (cubic and quartic) into quadratic terms. The resulting QUBO is implemented via a Hamilton spin and then mapped onto a hardware graph by encoding logical spins by chains of physical ones, giving additional overhead relative to qubits. We would like to point out that even before mapping the problem to the hardware, the increasing bandwidth of the QUBO coefficients according to Eq. (8) can be disadvantageous.

Focus on the factoring equations Tab. I. We will show that there are states where the objective grows as $\mathcal{O}(\log^3(n))$. To see this, consider the variable assignment $p_i = p_j = 1, n_i = 0$, and all carry variables set to zero which results in a cost of

$$\text{cost}(p, q, c, n) = \sum_{k=1}^{l} \left(\sum_i p_i q_{k-i} \right)^2 = \frac{1}{12} l^3 + \frac{1}{6} l. \tag{11}$$

This behavior is illustrated in Supplementary Fig. 8, which should be interpreted qualitatively and not quantitatively since the exact behavior depends on the specific mapping used to translate the higher-order terms into quadratic ones. For example, a cubic term like $\sigma_1\sigma_2\sigma_3$ can be decomposed into [21]

$$\begin{aligned} \sigma_1\sigma_2\sigma_3 \simeq 3 + \sigma_1\sigma_2 + \sigma_1\sigma_3 + \sigma_2\sigma_3 \\ + \sum_{i=1}^{3} (\sigma_a \sigma_i - \sigma_i) - 2\sigma_a. \end{aligned} \tag{12}$$

Where an auxiliary qubit 'a' is introduced and the equivalence $\simeq$ here should be understood as the existence of a one-to-one mapping between the ground-state manifolds in the sense that the states for the non-ancilla spins are identical. To lower-bound the spectral radius $\|H_1\|$ we try to find a highly excited state by realizing that misaligned auxiliary spins σ_a provide at least an additional energy of $+1$. We take this into account by replacing negative entries in Eq. (10) with positive ones $-c_{\text{out}} \mapsto c_{\text{out}}$ and $-n_k \mapsto n_k$. This results in the lower limit shown in Supplementary Fig. 8.

This discussion is evident when comparing the parity implementation to the standard multiplication table approach wherein the quantity $\max_s \|\dot{H}(s)\|$ scales favorably in the parity case. This is due to the constant interactions in Eqs. (9, 10) introduced in the main text and Supplementary Note 4. compared to increasing interaction strengths in the standard multiplication-table approach [8].

The big unknown is the minimum spectral gap during the annealing sweep Eq. (8). Although the number of physical qubits scale quadratically in the problem size, the smallest instance of a 2bit × 2bit parity multiplier requires 26 qubits and 66 qubits in the 3bit × 3bit case. Therefore, a scaling analysis of the minimal gap is outside the scope of this work. However, as already mentioned in the main text, the mapping of the QUBO to an annealer introduces roughly an additional square overhead in terms of qubits, as shown in Supplementary Fig. 1. Factoring large numbers quickly becomes a challenge as embedding monotonically decreases the performance of the annealing algorithm. This embedding effort in space and the mentioned improved spectral properties of the final Hamiltonian are our main arguments for the superiority of our approach over the standard approach.

We like to end this section with a final comment. Theoretical, there exists an adiabatic implementation capable to solve the factoring problem with polynomial resources in space and time. This is due to the polynomial equivalence of the gate model and the model of adiabatic computation established in Ref. [22]. The (time) efficiency of Shor's algorithm is preserved by this mapping,

although the final Hamiltonians in [22] are in general non-stoquastic and nonlocal. The work in [23] strengthened this result to a 2D construction with 2-local nearest-neighbor interactions. All of these circuit-to-Hamilton constructions are of theoretical interest rather than practical use, although they are polynomial in qubit numbers, they require enormous overhead. Here in this work, we focused more on minimizing spatial resources, e.g., number of qubits and type of interactions. In addition, in contrast to the work in [22, 23], our final Hamiltonian is a purely stochastic one. Up to this point, it is unclear whether non-stoquasticity is needed in order to guarantee a Shor-like time scaling, but it has been shown: Adding non-stoquastic terms can enhance the efficiency of quantum annealing by reducing a first-order quantum phase transition in the simple transverse-field case to a second-order transition [12].

VIII. SUPPLEMENTARY NOTE 8: REALISTIC IMPLEMENTATION

As shown in the last section, the desired ground state can be generated by a variety of different methods. Moreover, the problem could be implemented on different platforms, some of which are tailored to a specific algorithm. The bottleneck in implementing a single parity constraint is clearly the three- or four-body terms $\sigma_z^1\sigma_z^2\sigma_z^3(\sigma_z^4)$. Different proposals implement these constraints differently. Some of them introduce auxiliary qubits to decompose the higher-order terms into quadratic terms, as shown in Eq. (12). For example, Ref. [21] introduces a decomposition of a four-body term into two three-body terms and finally into a two-body interaction - introducing three auxiliary qubits. Another "odd parity" reduction, as presented in Ref. [24], introduces only a single auxiliary qubit, at the expense of a non-planar embedding compared to the previous planar embedding of Ref. [21]. Therefore, any platform that natively has tunable single-body interactions σ_z and pairwise interactions of the form $\sigma_z \otimes \sigma_z$ is suitable for implementing a parity-based design. This includes platforms such as superconducting qubits, e.g., transmon or flux qubits [25], ultracold atoms such as ultracold neutral alkali atoms [26], systems with trapped ions, quantum dots or with impurities in solid state crystals such as NV centers. Two concrete suggestions are presented in the context of superconducting transmon qubits in [21] or arrangements of Rydberg atoms in [27]. Common to all these proposals is that the decomposition entails an additional overhead, since each three- and four-body constraint is implemented via auxiliary qubits, which are not considered in Eq. (11) from the main text. However, suggestions that are basically overhead-free have recently been presented, which we would like to investigate in more detail here.

A. Rydberg parity-QAOA

As presented in [28], neutral atoms, e.g., Rubidium (^{87}Rb) trapped in tweezer arrays, laser coupled to highly excited Rydberg states, offer a promising platform to solve the combinatorial optimization problems. This proposal builds on the parity structure of the LHZ scheme [29, 30] and implements the Quantum Approximation Optimization Algorithm (QAOA) [19]. Let $H_{\mathrm{probl}} = H_z + H_c$ be a decomposition of the problem spin Hamiltonian H_{probl} into local fields denoted by H_z and H_c and all higher orders contain conditions associated with parity constraints.

For the implementation of the key component $U_c = e^{-i\gamma H_c}$, the authors in Ref. [28] propose a simple adiabatic protocol in the form of two laser pulses, coupling the computational basis states $|\downarrow\rangle$ and $|\uparrow\rangle$ with the Rydberg state $|r\rangle$. During and in-between these pulses, phases are accumulated as a result of the van-der-Waals interaction, which can be adjusted s.t. states of even parity gain the phase factor $e^{i\gamma}$ while odd parity state gains a factor of $e^{-i\gamma}$. This ultimately implements the action of the phase gate U_c.

The biggest difference between our proposal for parity factorization and the setting presented in [28] is the geometry itself. While the proposal is immediately applicable in the planar case, the three-layer construction discussed in Sec. "Three-layer layout" is technically more challenging to implement. The work in [28] focuses on a lattice of 2×2 plates in which they preferentially displace every other atom to arrange them in a tetrahedral configuration. This means that the distance between all four atoms participating in a plaquette is pairwise equal. This is easy to implement in the experiment: State-of-the-art tweezer experiments can pick up to 100 individually addressable atoms and arrange them deterministically in any 3D pattern [31].

As the authors point out in [28], the rearrangement was only done for convenience, non-tetrahedral plaquettes are viable at the expense of controlling an extra phase. This works as long as the atoms involved in each plaquette are close together, which we have ensured by construction in the factorization model. Finally, the presented work [28] only discusses 4-body plaquettes, but the same technique is easily applicable to 2-body or 3-body terms. As they further point out, the implementation of the entire many-body phase gate $e^{-i\gamma H_c}$ can be parallelized: To avoid crosstalk between the plaquettes, they are illuminated in a series of rounds. In each round, a set of non-overlapping and non-adjacent plaques are lit simultaneously. A constant number of illumination rounds is therefore sufficient, i.e., independent of the system size. This discussion shows that a QAOA-based implementation of the parity factoring proposal on a Rydberg setting is technically feasible and achievable within existing experimental techniques. Furthermore, the performance of parity-based QAOA could be improved by exploring different driver Hamiltonians as shown by [32]. Concluding

this section, we would like to point out that this is not the first QAOA proposal for factoring. Reference [33, 34] proposed and analyzed a Variational Quantum Factoring (VQF) algorithm based on QAOA.

B. Superconducting parity gates via KPOs:

Yet another promising implementation is based on superconducting Kerr Parametric Oscillators (KPOs). A single KPO is similar to a swing where a pendulum is driven by modulating the natural frequency, e.g., by changing the position of the center of mass (parametric drive). A KPO exhibits a bifurcation that allows binary Ising spins to be represented as two coherent states $|\pm\alpha\rangle = |\pm\sqrt{p/K}\rangle$. In addition, a KPO can generate non-classical superposition states, such as $\propto (|\alpha\rangle \pm |-\alpha\rangle)$ via a quantum adiabatic bifurcation. Combinatorial optimization problems can be addressed by mapping an Ising model onto a KPO network by introducing a non-dissipative linear coupling between the KPOs [35]. The ground state of the Ising model in such a quantum bifurcation machine (QbM) is prepared by a quantum adiabatic evolution increasing pump rate adiabatically. As suggested in Ref. [35], superconducting circuits with Josephson junctions are promising for the implementation of KPOs. The simplest model for a KPO is a frequency tunable transmon qubit, i.e., a superconducting qubit consists of a resonator with anharmonicity. Instead of a purely inductive element, a transmon qubit consists of a capacitor and a Josephson junction (JJ). The parametric modulation is implemented by replacing the Josephson junction with a dc-SQUID (a loop with two identical Josephson junctions), where the critical current can be controlled by the magnetic flux bias through the dc-SQUID.

To solve generic Ising problems on QbMs, Ref. [36] proposed to build on the LHZ scheme [29] and show how all-to-all spin couplings are generated by a two-dimensional array of KPOs by effectively introducing local four-body interactions. Remarkably, the four-body constraint for KPOs is easily implemented by four-wave mixing in a single Josephson junction coupled to four neighboring KPOs. When the four KPOs have different resonance frequencies fulfilling a four-photon resonance condition $\omega_1 + \omega_2 = \omega_3 + \omega_4$ and if the resonators are detuned to each other, the central JJ induces coupling of the form $-C(a_1^\dagger a_2^\dagger a_3 a_4 + \text{h.c.})$ in the rotating-wave approximation. These result in the four-body constraint in the coherent state basis. This four-body interaction is an always-on coupling and its strength C is determined by the JJ nonlinearity.

Since our Hamiltonian factorization is built from parity plaquettes, the proposed couplers are of immediate use to build a KPO-based superconducting factorization device. As mentioned in section "Rydberg parity-QAOA", the main difference between the LHZ proposal and the three-layered factorization scheme is its non-planar geometry. The planar case though is directly implementable within the scope of the proposal presented in Ref. [36]. Therefore, in the remaining part of the section, we discuss the challenges to overcome to implement the three-layer proposal introduced in the main text Sec. "Three-layer layout".

One of the characteristics of a superconducting qubit like Transmon/Flux or Fluxonium is that they are essentially macroscopic induction loops interrupted by Josephson junction(s) and these qubit body loops can be stretched and moved as needed. This gives a lot of design freedom when manufacturing a superconducting quantum chip. This is most easily done when the interaction graph itself is planar, without the need to cross superconducting wires.

Commercially available annealers like the DWave series are based on non-planar hardware graphs [37, 38]. In fact, it has been shown that non-planarity is essential when aiming to solve NP-hard problems [39]. These annealers are built via a monolithic manufacturing process, creating planarized multilayer structure. A small number of metal layers is sufficient to implement qubits, couplers, and readout components according to the Chimera or Pegasus topology [40] on a single chip. These layouts implement qubits via horizontal and vertical loops of wires which couple in pairs when they cross. However, with current manufacturing techniques, the price of monolithic manufacturing is a severe disadvantage for qubit coherence.

In our case, dealing with a more complex hardware graph, we think the 3D integration approach is promising. While 3D integration is well developed for commercial electronics, the work in Ref. [41] analyzes the approach in the setting of superconducting qubits. They find it is possible to separate qubit circuitry from control and readout circuitry while maintaining high qubit coherence. The transition to the third dimension facilitates qubit addressability, enables efficient connection routing to large 2D arrays, allows for more compact qubit-qubit coupling geometries, and offers significantly improved connectivity beyond nearest-neighbor interactions. The ability to stack multiple chips on top of each other could be the key to realizing our three-layer proposal in connection with the ability to natively implement 3- and 4-body terms. Hence, we consider the on-chip implementation of our factorization-dependent connectivity as superconducting circuits as a challenging but doable technical hurdle.

The design of higher-order couplers is an ongoing and active area in the development of superconducting circuits. As an example, we refer to Ref. [42], which presents a 4-local coupler based on flux qubits found by automated circuit design. Further results will improve the quality of these couplers in terms of circuit complexity, noise sensitivity, or other performance measures.

SUPPLEMENTARY REFERENCES

[1] A. Lucas, Frontiers in Physics **2**, 5 (2014).
[2] T. Kadowaki and H. Nishimori, Physical Review E **58**, 5355–5363 (1998).
[3] Y. Susa, Y. Yamashiro, M. Yamamoto, I. Hen, D. A. Lidar, and H. Nishimori, Phys. Rev. A **98**, 042326 (2018).
[4] P. Hauke, H. G. Katzgraber, W. Lechner, H. Nishimori, and W. D. Oliver, Reports on Progress in Physics **83**, 054401 (2020).
[5] J. Kempe, A. Kitaev, and O. Regev, SIAM Journal on Computing **35**, 1070 (2006), https://doi.org/10.1137/S0097539704445226.
[6] E. Farhi, J. Goldstone, S. Gutmann, and M. Sipser, "Quantum computation by adiabatic evolution," (2000), arXiv:0001106 [quant-ph].
[7] R. Mengoni, D. Ottaviani, and P. Iorio, "Breaking rsa security with a low noise d-wave 2000q quantum annealer: Computational times, limitations and prospects," (2020), arXiv:2005.02268 [quant-ph].
[8] S. Jiang, K. A. Britt, A. McCaskey, T. Humble, and S. Kais, Scientific Reports **8** (2018).
[9] M. Lanthaler and W. Lechner, New Journal of Physics **23**, 083039 (2021).
[10] T. J. Schaefer, in *Proceedings of the Tenth Annual ACM Symposium on Theory of Computing*, STOC '78 (Association for Computing Machinery, New York, NY, USA, 1978) p. 216–226.
[11] M. S. Könz, G. Mazzola, A. J. Ochoa, H. G. Katzgraber, and M. Troyer, Phys. Rev. A **100**, 030303 (2019).
[12] H. Nishimori and K. Takada, Frontiers in ICT **4** (2017), 10.3389/fict.2017.00002.
[13] Y. Susa, Y. Yamashiro, M. Yamamoto, and H. Nishimori, Journal of the Physical Society of Japan **87** (2018), 10.7566/JPSJ.87.023002.
[14] A. Hartmann and W. Lechner, Physical Review A **100** (2019), 10.1103/physreva.100.032110.
[15] D. Guéry-Odelin, A. Ruschhaupt, A. Kiely, E. Torrontegui, S. Martínez-Garaot, and J. G. Muga, Rev. Mod. Phys. **91**, 045001 (2019).
[16] A. Hartmann and W. Lechner, New Journal of Physics **21**, 043025 (2019).
[17] A. Peruzzo, J. McClean, P. Shadbolt, M.-H. Yung, X.-Q. Zhou, P. J. Love, A. Aspuru-Guzik, and J. L. O'Brien, Nature Communications **5**, 4213 (2014).
[18] J. Tilly, H. Chen, S. Cao, D. Picozzi, K. Setia, Y. Li, E. Grant, L. Wossnig, I. Rungger, G. H. Booth, and J. Tennyson, "The variational quantum eigensolver: a review of methods and best practices," (2021), arXiv:2111.05176 [quant-ph].
[19] E. Farhi, J. Goldstone, and S. Gutmann, "A quantum approximate optimization algorithm," (2014), arXiv:1411.4028 [quant-ph].
[20] T. Albash and D. A. Lidar, Rev. Mod. Phys. **90**, 015002 (2018).
[21] M. Leib, P. Zoller, and W. Lechner, Quantum Science and Technology **1**, 015008 (2016).
[22] D. Aharonov, W. van Dam, J. Kempe, Z. Landau, S. Lloyd, and O. Regev, in *45th Annual IEEE Symposium on Foundations of Computer Science* (2004) pp. 42–51.
[23] R. Oliveira and B. M. Terhal, Quantum Info. Comput. **8**, 900 (2008).
[24] A. Rocchetto, S. C. Benjamin, and Y. Li, Science Advances **2** (2016), 10.1126/sciadv.1601246.
[25] P. Krantz, M. Kjaergaard, F. Yan, T. P. Orlando, S. Gustavsson, and W. D. Oliver, Applied Physics Reviews **6**, 021318 (2019), https://doi.org/10.1063/1.5089550.
[26] M. Saffman, Journal of Physics B: Atomic, Molecular and Optical Physics **49** (2016), 10.1088/0953-4075/49/20/202001.
[27] A. Glaetzle, R. Bijnen, P. Zoller, and W. Lechner, Nature Communications **8** (2016), 10.1038/ncomms15813.
[28] C. Dlaska, K. Ender, G. B. Mbeng, A. Kruckenhauser, W. Lechner, and R. van Bijnen, Phys. Rev. Lett. **128**, 120503 (2022).
[29] W. Lechner, P. Hauke, and P. Zoller, Science Advances **1** (2015), 10.1126/sciadv.1500838.
[30] K. Ender, R. ter Hoeven, B. E. Niehoff, M. Drieb-Schön, and W. Lechner, "Parity quantum optimization: Compiler," (2021), arXiv:2105.06233 [quant-ph].
[31] D. Barredo, V. Lienhard, S. de Léséleuc, T. Lahaye, and A. Browaeys, Nature **561**, 79 (2018).
[32] K. Ender, A. Messinger, M. Fellner, C. Dlaska, and W. Lechner, PRX Quantum **3**, 030304 (2022).
[33] E. Anschuetz, J. Olson, A. Aspuru-Guzik, and Y. Cao, in *Quantum Technology and Optimization Problems*, edited by S. Feld and C. Linnhoff-Popien (Springer International Publishing, Cham, 2019) pp. 74–85.
[34] A. H. Karamlou, W. A. Simon, A. Katabarwa, T. L. Scholten, B. Peropadre, and Y. Cao, npj Quantum Information **7**, 156 (2021).
[35] H. Goto, Scientific Reports **6**, 21686 (2016).
[36] S. Puri, C. K. Andersen, A. L. Grimsmo, and A. Blais, Nature Communications **8**, 15785 (2017).
[37] P. I. Bunyk, E. M. Hoskinson, M. W. Johnson, E. Tolkacheva, F. Altomare, A. J. Berkley, R. Harris, J. P. Hilton, T. Lanting, A. J. Przybysz, and J. Whittaker, IEEE Transactions on Applied Superconductivity **24**, 1 (2014).
[38] K. Boothby, C. Enderud, T. Lanting, R. Molavi, N. Tsai, M. H. Volkmann, F. Altomare, M. H. Amin, M. Babcock, A. J. Berkley, C. B. Aznar, M. Boschnak, H. Christiani, S. Ejtemaee, B. Evert, M. Gullen, M. Hager, R. Harris, E. Hoskinson, J. P. Hilton, K. Jooya, A. Huang, M. W. Johnson, A. D. King, E. Ladizinsky, R. Li, A. MacDonald, T. M. Fernandez, R. Neufeld, M. Norouzpour, T. Oh, I. Ozfidan, P. Paddon, I. Perminov, G. Poulin-Lamarre, T. Prescott, J. Raymond, M. Reis, C. Rich, A. Roy, H. S. Esfahani, Y. Sato, B. Sheldan, A. Smirnov, L. J. Swenson, J. Whittaker, J. Yao, A. Yarovoy, and P. I. Bunyk, "Architectural considerations in the design of a third-generation superconducting quantum annealing processor," (2021), arXiv:2108.02322 [quant-ph].
[39] F. Barahona, Journal of Physics A: Mathematical and General **15**, 3241 (1982).
[40] P. I. Bunyk, E. M. Hoskinson, M. W. Johnson, E. Tolkacheva, F. Altomare, A. J. Berkley, R. Harris, J. P. Hilton, T. Lanting, A. J. Przybysz, and et al., IEEE Transactions on Applied Superconductivity **24**, 1–10 (2014).
[41] D. Rosenberg, D. Kim, R. Das, D. Yost, S. Gustavsson, D. Hover, P. Krantz, A. Melville, L. Racz, G. O. Samach,

S. J. Weber, F. Yan, J. L. Yoder, A. J. Kerman, and W. D. Oliver, npj Quantum Information **3**, 42 (2017).
[42] T. Menke, F. Häse, S. Gustavsson, A. J. Kerman, W. D. Oliver, and A. Aspuru-Guzik, npj Quantum Information **7**, 49 (2021).
